# Speak or shout? Nonverbal vocalizations promote rapid detection of emotions in vocal communication

**DOI:** 10.1371/journal.pone.0327529

**Published:** 2026-01-08

**Authors:** Marc D. Pell, Haining Cui, Yondu Mori, Xiaoming Jiang

**Affiliations:** 1 School of Communication Sciences and Disorders, McGill University, Montréal, Québec, Canada; 2 Institute of Linguistics, Shanghai International Studies University, Shanghai, China; Macquarie University, AUSTRALIA

## Abstract

Human vocal expressions of emotion can be expressed nonverbally, through vocalizations such as shouts or laughter, or speakers can embed emotional meanings in language by modifying their tone of voice (“prosody”). Is there evidence that nonverbal expressions promote “better” (i.e., more accurate, faster) recognition of emotions than speech, and what is the impact of language experience? Our study investigated these questions using a cross-cultural gating paradigm, in which Chinese and Arab listeners (n = 25/group) judged the emotion communicated by acoustic events that varied in duration (200 milliseconds to the full expression) and form (vocalizations or prosody expressed in listeners’ native, second or foreign language). Accuracy was higher for vocalizations overall, but listeners were markedly more *efficient* to form stable categorical representations of the speaker’s emotion from vocalizations (M = 417ms) than native prosody (M = 765ms). Language experience enhanced recognition of emotional prosody expressed by native/ingroup speakers for some listeners (Chinese) but not all (Arab), emphasizing the dynamic interplay of socio-cultural factors and stimulus quality on prosody recognition which occurs over a more sustained time window. Our data show that vocalizations are functionally suited to build robust, rapid impressions of a speaker’s emotion state unconstrained by the listener’s linguistic cultural background.

## Introduction

Human vocal expressions of emotion take two principal forms: they can be expressed nonverbally through laughter, shouts, or other emotionally relevant *vocalizations*; or speakers can embed emotional meaning in language by modifying suprasegmental acoustic features of their “tone of voice” (*speech prosody*) [1]. Understanding what is shared and what is distinct about these two communication subsystems—in terms of their expressive form, social functions, neurocognitive underpinnings, and impact on listeners—has been the focus of recent work [2–4]. Notably, some researchers argue that vocalizations are functionally selected over speech to maximize accuracy and *efficiency* (i.e., speed of response) to detect emotions, particularly in aversive situations [5,6]. The extent to which communicating emotions in speech is dependent on language experience has also been raised [7]. Our study addresses these debates by comparing emotion recognition from vocal events that varied systematically in *form* (vocalizations vs. prosody expressed in a listener’s native, second or foreign language) and *exposure duration* (stimuli lasted from 200 milliseconds to full expressions). In this way, our design allows new insights about the *time course* of processes that lead to emotion recognition in the vocal channel and how they are shaped by stimulus properties (acoustic duration, linguistic characteristics).

### One channel, two forms of expression

Emotional vocalizations and emotional speech prosody share much in common; both consist of dynamic acoustic patterns that vary in perceived pitch, energy, quality, and temporal sound properties that progressively gain significance over time [8]. There is evidence that both forms of expression exploit a common set of acoustic features that allow listeners to differentiate and attribute a range of discrete emotion states to the speaker [4,9]. These mappings allow ‘basic’ emotions such as anger, fear, happiness and many other meanings to be successfully detected in the voice at levels far exceeding chance, both within and across cultural boundaries [10–15].

Still, it seems obvious that saying, *“I don’t know what to do”* in a sad voice, or beginning to sob uncontrollably, are quite different exemplars of “sadness” that will have distinct perceptual and interpersonal effects [16]. At the production stage, vocalizations allow greater flexibility and acoustic variability in their expression than speech-embedded emotions, which are constrained by simultaneous demands on linguistic production [17,18]. As a result, nonverbal signals are believed to capture preferential attention [19,20] and encode emotions with greater perceptual clarity (i.e., reduced sensory ambiguity), promoting higher recognition rates than prosody when formally measured in experimental tasks [2,4]. Vocalizations also appear to be processed with greater automaticity than speech [3,21,22], promoting fast and efficient recognition of emotions that are minimally hampered by conscious deliberation or simultaneous cognitive load [23]. The apparent primacy of emotional vocalizations in perception could reflect the fact that nonverbal signals emanate from a biologically primitive, reflexive call system adapted by humans (and other species) for survival, whereas emotions in speech are cognitively mediated and under significant voluntary control [24–26]. As such, processes of socialization—including cultural preferences, language familiarity, and other forms of learning—are believed to exert stronger effects on how emotional prosody is recognized in the vocal channel than nonverbal expressions [10,13,27,28].

### On the time course of vocal emotion recognition

But how does the form of vocal expressions alter the *path* to emotion recognition over time, and in what way(s) do these operations depend on listener experience (e.g., previous exposure or familiarity with particular event types)? Surprisingly, direct comparisons of vocalizations and speech prosody are still few. One way to infer how and *when* vocal emotions are recognized is to measure event-related brain potentials (ERPs) evoked by a vocal stimulus. When listeners are presented prosody in their native language, there is evidence that the brain differentiates discrete emotional qualities of speech beginning 200 milliseconds post-onset of an utterance [3,29], at least in sufficient terms to facilitate deeper cognitive analysis of the event’s contextual meaning at later timepoints (see [30] for a recent discussion). ERP studies that have presented vocalizations tend to report even earlier cortical responses, with emotion-related changes in activity often beginning 100ms post-onset of vocalizations [21,31]. These studies suggest that perceptual and cognitive operations for ‘recognizing’ emotion from voices proceed rapidly, but that vocalizations are differentiated at *an earlier time point* by the neurocognitive system than prosody. This claim is reinforced by an ERP experiment that compared the two expression types directly: vocalizations of anger, sadness, and happiness produced earlier and more qualitatively distinct brain responses in listeners than when these emotions were expressed prosodically in native-like pseudo-utterances [3].

Another way to illuminate the time course of vocal emotion recognition is by “gating” auditory stimuli, i.e., presenting time-limited excerpts of a vocal stimulus (e.g., the first 200 or 400 milliseconds), to gauge their effects on perception and behaviour. Using gated prosodic stimuli in a novel voice-face priming paradigm, Pell and colleagues [32,33] concluded that English listeners require at least 400ms of speech exposure to prime decisions about an emotionally-related face, suggesting that discrete meanings of prosody are activated and implicitly “recognized” from ~400ms of acoustic information (see also [34]). Interestingly, when emotions were expressed in a foreign language (Arabic), English listeners required prolonged exposure to the prosodic input (>600ms) for priming to occur [35]. These findings suggest that language familiarity is a critical factor governing the recognition of speech-embedded emotions and alters its recognition time course [36].

Other experiments have probed these questions using an auditory gating paradigm adapted from [37], whereby participants judge vocal emotion expressions presented in time- or structure-based increments which always increase in duration over the course of the study (e.g., participants render forced-choice emotion judgements after hearing the first 100ms, 200ms, or 300ms of the same event). This approach allows researchers to estimate how much acoustic input listeners need to form stable categorical representations of emotion based on a specific stimulus duration, by comparing “gate-to-gate” increases in recognition accuracy, while ensuring that acoustic details that promote recognition always build up incrementally from shortest to longest event duration. Studies that have gated emotional prosody in languages such as English, Hindi, or Swedish show that speech-embedded emotions are accurately recognized at notably different latencies or “speeds” [36,38–41]. While patterns vary from study to study due to differences in how gates were defined, most gating studies conclude that listeners need at least 400–500ms of emotional prosody for recognition to begin to stabilize (“emotion identification point”), meaning that participants can identify the target meaning at this timepoint and do not change their mind at longer exposures [38,39,41]. The time course for recognizing specific emotions from prosody varies markedly: anger, sadness and (sometimes) fear tend to be isolated earliest from ~500–800ms of acoustic information, whereas other emotions (e.g., happiness, disgust, interest) often require 1–2 seconds of speech cues to identify [36,39,41]. Moreover, certain speech-embedded emotions, particularly happiness, seem to depend heavily on linguistic structure and can only be isolated when listeners integrate acoustic cues provided toward the end of an utterance [10,40,42].

Subsequent experiments that have gated vocalizations report that stable emotion representations can typically be formed after hearing ~250–350ms of acoustic input, much earlier than for prosody [43–45]. Castiajo & Pinheiro (2019) observed large increases in target hit rates for 10 different emotional vocalizations that lasted between 200–300ms, with fastest recognition of amusement (laughter) and slowest recognition of fear. However, this literature is again marked by many methodological differences related to the number and type of emotions studied and the way that vocalizations were gated for presentation (e.g., 33ms vs. 100ms intervals). Moreover, studies have not gated vocalizations and speech-embedded emotions in a unitary manner for evaluation by the same participants to directly compare if the time course of recognition differs as a function of their expressive form. To address these issues, our study undertook a direct test of how emotion recognition unfolds from vocalizations vs. speech prosody using the gating paradigm to determine whether nonverbal stimuli are recognized “more efficiently” (i.e., at an earlier timepoint) than speech prosody, as suggested by recent findings [3,5].

As a secondary goal, we sought to shed light on how *familiarity* with a language influences the recognition of speech-embedded emotions. According to Dialect theory, socially-constructed forms of emotional communication, such as prosody, are shaped by cultural “styles” which provide listeners an advantage to recognize emotions expressed by native (‘ingroup’) speakers [46]. In the only emotional gating study to implement a cross-cultural design, Jiang and colleagues [36] required groups of English Canadian and Indian listeners to recognize four emotions (anger, fear, happiness, sadness) from both English and Hindi pseudo-utterances gated to six exposure durations (200ms, 400ms, 500ms, 600ms, 700ms, full utterance). Results showed that recognition accuracy was significantly higher for native prosody in each group (ingroup advantage), emphasizing the importance of language experience on emotional prosody recognition [7]. In addition, emotion identification points occurred earlier in time for native prosody, irrespective of whether the non-native language was considered foreign to the listener (Canadians judging Hindi) or the listener’s second language/L2 (Indians judging English). Interestingly, the Indian participants’ ability to recognize emotions in L2-English was positively associated with their English proficiency level, although this relationship is not consistently reported elsewhere in the literature (cf. [47–49]. These findings motivate a deeper look at the impact of language familiarity—i.e., whether emotions are expressed in a listener’s native, second, or a completely foreign language—on both the accuracy and time course of emotional prosody recognition [7,15].

### Aim of the current study

Here, we investigated how emotions are recognized from vocalizations versus speech prosody using an adapted version of Jiang et al.’s [36] cross-cultural gating paradigm. Our new design simultaneously allowed us to evaluate the impact of language familiarity on recognition performance within and between two distinct groups: native speakers of Mandarin-Chinese and Arabic, all of whom were proficient second-language speakers of English. Based on above, we predicted that recognition accuracy would be higher and stabilize at earlier timepoints for vocalizations than for native prosodic expressions in each group; emotion-specific recognition trajectories in each condition were likely to vary, but these patterns should be more similar between groups when judging vocalizations than emotional prosody, which is shaped by linguistic and cultural variables to a greater extent [7]. For speech-embedded emotions, it was expected that language familiarity would enhance accuracy and speed of emotional prosody recognition when each listener group judged their native language, consistent with the ingroup advantage (native > foreign and L2-English). We further speculated that high proficiency in L2-English would enhance emotional prosody recognition over foreign prosody [36,47,49], although there is no clear precedent for comparing these measures simultaneously in native, L2, and foreign language contexts.

## Materials and methods

### Participants

Fifty young adults judged all vocal expressions presented in the study; assuming medium effect sizes, this sample size achieves power exceeding.95 to detect differences in event type if mixed ANOVAs had been employed (this estimation may be considered the lower bound of actual power achieved in the study using linear mixed effects models, [50]). Participants were recruited from the greater Montréal region on the basis of speaking Arabic (n = 25, 15F/10M, Mean Age = 21.5 ± 3.4, Mean Education = 15.6 years ± 3.8) or Mandarin-Chinese (n = 25, 18F/7M, Mean Age = 22.2 ± 4.2, Mean Education = 15.6 years ± 2.4) as a native language, and for having high proficiency in English as a second language. Participants were students or recent immigrants who moved to Canada as adults and had lived in Montréal for less than five years (Median duration in Canada: Arab group = 18 months, range = 4–58 months; Chinese group = 12 months, range = 1–24 months). Most participants arrived in Canada between the ages of 19–21. Arab participants were born and raised in several Arabic-speaking countries (Syria, Jordan, Bahrain), although the majority (20/25) spoke variants of Levantine Arabic. Chinese participants were born and raised in different regions of mainland China (Shanghai, Beijing, Shenzhen).

All participants learned English in school as a second language (L2-English) from an early age (Mean Age of English onset: Arab = 5.7 years ± 3.4; Chinese = 8.1 years ± 3.3). L2-English proficiency was characterized through a series of self-report measures gathered at the onset of the study; all participants in each group rated their ability to speak and listen in English as high (group means ranged between 7.7–9.5 on a 10-point proficiency scale, Table 1). Many participants in each group knew additional languages (e.g., French, Farsi, Cantonese). To enter the study, it was verified that no participant had any knowledge of the language designated as “foreign” for that group in the experiment (i.e., Arabic for Chinese participants; Chinese for Arab participants). All participants reported normal hearing. Recruitment began on 07/21/2016 and ended on 05/26/2017. Voluntary written consent was obtained prior to the study, which was approved by the Faculty of Medicine Institutional Review Board, McGill University.

**Table 1:** Demographic characteristics of participants in the Chinese and Arab groups.

	Chinese Group (n = 25)		Arab Group (n = 25)	
Variable	Mean	SD	Mean	SD
Sex (f/m)	18/7	--	15/10	--
Age at testing (years)	22.2	4.2	21.5	3.4
Age of arrival to Canada (years)	21.2	8.1	19.8	4.2
Time in Canada (months)	12.4	4.6	21.8	16.2
Education (years)	15.0	2.4	15.6	3.8
Age of L2-English onset (years)	8.1	3.3	5.8	3.4
Self-reported L2-English Proficiency (scale 1–10)				
Listening	8.6	1.3	9.5	0.8
Speaking	7.7	1.5	8.8	1.6
Reading	8.2	1.4	9.4	0.9
Writing	7.2	1.9	8.8	1.2

### Materials

**Fig 1:**
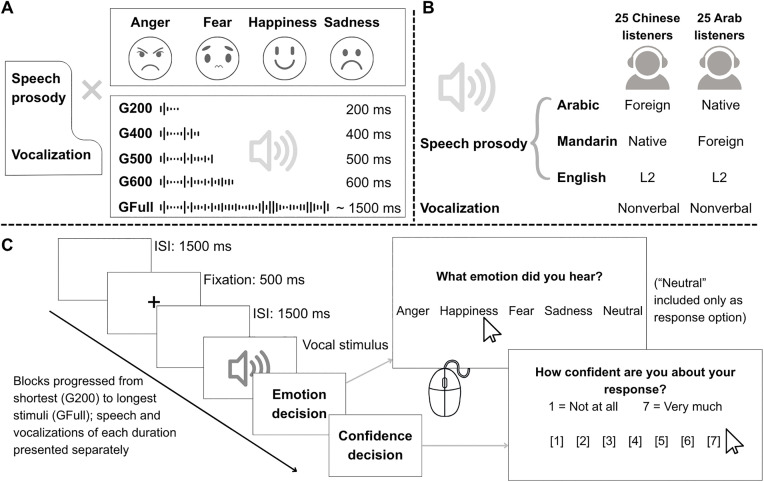
Experimental design and procedure. **(A)** Stimuli expressed one of four emotions (anger, fear, happiness, sadness) through speech prosody or vocalizations. Each stimulus was cut from vocal onset and presented in five “gate” (G) duration blocks: 200ms, 400ms, 500ms, 600ms, or the full expression (GFull). **(B)** Language familiarity conditions as a function of event type for Mandarin and Arabic perceiver groups. **(C)** Trial sequence showing timing and two mouse-click responses (emotion choice and confidence rating). Blocks always presented stimuli in order of increasing acoustic duration, separately for speech and vocalizations.

The stimuli were digital auditory recordings of vocally expressed emotion ~1–3 seconds in duration, taken from established inventories used actively in the literature [15,27,51–53]. All recordings were elicited in laboratory settings using lay speakers/actors and then perceptually validated by different groups of listeners. The study included two distinct event types: nonverbal *vocalization*s and speech-embedded emotional expressions (henceforth referred to as *vocalization* and *speech prosody*). Speech prosody could be further divided by language of expression (Arabic, Mandarin, English), which differed in familiarity to each perceiver group (defined as native, L2-English, or foreign). Vocalizations and prosody in each language communicated one of four discrete emotions: anger, fear, sadness, or happiness (Fig 1A,1B).

Vocalizations took the form of growls or shouts (anger), cries (fear), sobbing or wailing (sadness), and laughter or contentment sounds (happiness). All stimuli were nonverbal in nature, although some resembled sustained vowels or nasal sounds found in most languages (“aah” or “mmm”). Stimuli in the speech prosody condition were emotionally-inflected pseudo-utterances composed of 7–11 syllables/characters produced by native speakers of Arabic, Mandarin-Chinese, or English [15,53]. Pseudo-utterances, which mimic linguistic properties of a language while restricting emotion to the speaker’s prosody, have been used in previous gating studies [36,38,39] and broadly in the prosody literature (see [8] for an overview). Pseudo-speech stimuli in each of the three language conditions were constructed, recorded, and perceptually validated using virtually identical procedures, but involving native speakers-listeners of only the target language (see [53]) for details). To mimic the prosody condition, vocalizations were selected from three separate corpora produced by French Canadian, European Portuguese, and British English speakers [27,51,52]. This decision was meant to increase generalizability of results to diverse speakers and allow finer selection of vocalizations that matched perceptual features of the speech stimuli to the extent possible.

Perceptual data from the original studies were used to select a controlled set of angry, sad, fearful and happy exemplars suitable for directly comparing the effects of vocal event type on the time course of emotion recognition. All selected vocalizations achieved high consensus about the target emotion according to the original study design (accuracy > 5 times chance). Vocalizations too brief for effective gating or much longer than the speech stimuli were excluded. In the case of vocalizations representing “happiness”, we noted that emotional meaning labels validated in the original inventories often varied (e.g., “pleasure”, “contentment”, “amusement”, “happiness”). As these terms likely refer to a broader range of positive emotions that can be communicated nonverbally than in speech [54], we initially selected two subsets of “happy” vocalizations to compare with speech prosody in an exploratory manner: happiness-*amusement*, characterized uniquely by laughter sounds; and happiness-*pleasure,* composed of vocal sounds of pleasure or contentment (e.g., “mmm”, “aah”). Ten unique vocalizations were selected for anger, fear, and sadness and 20 unique items for happiness (10 amusement, 10 pleasure). This totalled 50 vocalizations, produced by a variety of speakers (7–10 speakers/ emotion, half female, half male, 18 distinct voices in total). Selected vocalizations varied naturally in duration with a median of 1448ms (range = 715–2376ms) prior to gating.

Prosodic exemplars of each emotion were selected for one female and one male native speaker of each language, who each produced six distinct pseudo-utterances to convey each emotional target (anger, fear, sadness, happiness, 12 items/emotion). This process yielded 48 unique prosodic expressions/language (2 speakers x 4 emotions x 6 utterances). Items were again selected for having high emotion recognition rates when judged by a native listener group in the original study (minimum 3x chance accuracy based on a seven forced-choice task). In addition, items were chosen to mitigate gross differences in native emotion recognition accuracy *across language sets* to the extent possible (Mean emotional target recognition range by native listeners: Arabic = 55–74%, Mandarin = 64–82%, English = 64–80%). A total of 144 utterances (3 languages x 48 items) were selected for gating in the speech prosody condition. The speech stimuli were roughly similar in duration (Median = 1493ms, range = 834–2900ms) to items in the vocalization condition.

### Experimental design and procedures

#### Gate construction.

Vocalizations (n = 50) and speech prosody (n = 144) were edited using Praat speech analysis software to standardize the peak volume of all sound files (75dB) and to segment each stimulus into four additional gates which varied in duration. Each item was cut from its acoustic onset to isolate the initial 200ms, 400ms, 500ms, and 600ms of the stimulus, which were saved as separate.wav files and manually edited to eliminate any noise artefacts (clipping noise). This procedure yielded five gate conditions per stimulus, the final gate always being the original unedited sound; these gates are referred to as G200, G400, G500, G600, and GFull. The choice of gate durations was informed by emotion identification points reported in previous studies [39,43] as well as Jiang et al.’s [36] cross-cultural study which reported effects of language familiarity on prosody recognition in the 400–600ms latency range. The gating process created 250 distinct trials for presentation in the vocalization condition (50 items x 5 gates) and 720 trials in the speech condition (3 languages x 48 items x 5 gates).

#### Testing procedures.

Participants were tested in a quiet laboratory, individually or in small groups (2–4 people), seated at individual workstations. Listeners heard stimuli over volume adjustable headphones controlled by Superlab 5.0 presentation software (Cedrus, CA). Instructions about the experiment were first provided verbally in English. Participants were told that they would hear vocal sounds or utterances that could sound familiar but would not make sense; they were instructed to pay attention to the speaker’s voice to decide what emotion the sound conveys *and* how confident they felt about their decision. They were told that stimuli would begin short and would sound “cut off”, and that sounds would increase in duration over the experiment. Participants were explicitly instructed to choose the label that *best fits* their impression of the emotion being expressed or to guess when they were unsure. Once the study began, written instructions and all other features of the experiment (e.g., emotion labels) were only presented in the participant’s native language (Arabic or Mandarin).

Trials were divided and presented to participants in five separate blocks according to their acoustic duration, always starting with the shortest gate (G200) and ending with the full stimulus (GFull, Fig 1A–1C). This fixed presentation order is standard in auditory gating research to ensure that recognition at short exposure durations is not biased by hearing longer excerpts of the same stimuli first [37,39]. Within each gate duration block (e.g., G200, G400…), we further separated the speech and vocalization stimuli and placed them in different presentation blocks; the two vocal event types were never intermixed. In the speech condition, prosodic expressions in the three languages (Arabic, Mandarin, English) were fully intermixed. As participants judged stimuli cut to each gate duration, half of the participants in each group heard the prosodic stimuli first and half judged the vocalizations first. Individual items in each presentation block (e.g., G200 – vocalizations) were fully randomized. Each block always began with five practice trials.

Each trial began with an inter-stimulus interval (ISI) of 1500ms, a 500ms fixation cross, another 1500ms ISI, the auditory target, followed by a response screen. Participants clicked one of five emotion labels (anger, happiness, fear, sadness, neutral) displayed in the participant’s native language (for Mandarin: 高兴, 生气, 害怕, 难过, 中性; for Arabic, محايد, حزن, خوف, غضب, سعادة.). Immediately after, participants saw a new screen and rated their confidence in their judgment along a 7-point scale (1 = *not at all* to 7 = *very much*, Fig 1C). Although we did not present neutral vocal expressions in our study, a “neutral” response option was added in light of evidence that emotional voices are often recognized as neutral when acoustic exposure to the stimulus is brief [36,38,39]. The experiment took approximately 2.5 hours to complete. Given its length, frequent breaks were programed to reduce fatigue; a pause was inserted every ~30 trials within blocks and mandatory 5-minute breaks were imposed after judging stimuli at each gate duration). Participants received $25 CAD as compensation at the end of the study.

#### Statistical analysis.

Accuracy and latency of emotion recognition served as the two dependent measures of interest. Accuracy was estimated using Hu scores [55], the proportion of correct target responses assigned to each emotion adjusted for the number of items in each category and individual biases in category usage. Individual Hu scores were calculated for each emotion at each gate interval, separately for vocalizations and speech prosody judged in each language context. Recognition latency was estimated by calculating the Emotion Identification Point (EIP) for each item [39]. The EIP is the gate at which a participant correctly recognized the target meaning of a stimulus without changing their response at longer exposures of the same event, expressed in milliseconds (200ms, 400ms, 500ms, 600ms or the actual full event duration, ranging from 834–2900ms). Thus, EIPs considered a maximum of 250 datapoints per emotion/group for vocalizations and 300 judgements per emotion/group for each speech prosody condition. Items that did not lead to stable recognition by GFull were scored as errors and excluded from EIP calculations [39]. On average, 1642 observations (range = 1433–1838) contributed to the calculation of EIPs for each event type.

Linear mixed-effects models (LMM) were built to separately infer how accuracy (Hu score) and latency (EIP) measures were influenced by our experimental manipulations. Analyses were performed in R Studio (Version 4.2.2; http://cran.r-project.org) using the lme4 package [56] with Satterthwaite approximations method for providing degrees of freedom and F-statistics implemented in the *lmerTest* package [50]. Most LMMs included some combination of the fixed factors: perceiver Group (Chinese, Arab), vocal Event type (vocalization, native, L2-English, foreign), Emotion (anger, fear, happiness, sadness), and/or Gate duration (G200, G400, G500, G600, GFull), with Participant entered as a random factor. Emotion was also entered as a random effect in later models, along with variability by participant to allow for a focus on the broad role of vocal event type and language familiarity. Given that our stimuli (both vocalizations and speech) varied considerably in length, GFull duration (in milliseconds) was included as a covariate in models considering the EIP data to eliminate any potential effect of stimulus duration on the latency data. The *emmeans* package [57] with Tukey’s adjustments was used to control for multiple comparisons for all post hoc procedures.

## Results

Table 2 presents the unbiased accuracy rates (mean Hu score) at each stimulus duration, separately for the Chinese and Arab participants by event type and emotion. Table 3 shows the frequency/percentage of EIPs occurring at each stimulus duration across conditions, trials scored as errors (failure to recognize the target), and the mean EIPs for each emotion expressed in milliseconds (ms). The proportion of “neutral” responses assigned in each condition and the mean confidence ratings of each participant group when judging different forms of stimuli are furnished in S1 and S2 Tables. The datasets generated during and/or analysed for the current study are available in the Open Science Framework repository (https://osf.io/43udq/?view_only=d2b4a9cdbb9541f3b292f2a8c490616d).

**Table 2:** Recognition Accuracy – Unbiased hit rates (mean Hu scores) for Chinese and Arab participants by vocal event type, emotion, and gate duration.

	Chinese group (*n* = 25)					Arab group (*n* = 25)				
Emotion/Gate	G200	G400	G500	G600	GFull	G200	G400	G500	G600	GFull
Vocalization										
Anger	.60	.61	.60	.58	.63	.68	.70	.76	.77	.76
Fear	.35	.53	.53	.59	.71	.32	.57	.63	.62	.71
Happiness-amusement	.25	.50	.54	.52	.56	.34	.61	.59	.57	.57
Happiness-pleasure	.03	.05	.07	.10	.18	.04	.09	.13	.17	.22
Sadness	.32	.46	.47	.56	.73	.28	.52	.59	.64	.82
English prosody										
Anger	.15	.22	.32	.34	.49	.23	.40	.44	.55	.72
Fear	.09	.10	.14	.18	.27	.12	.22	.26	.30	.59
Happiness	.03	.09	.13	.19	.33	.05	.13	.20	.23	.54
Sadness	.09	.17	.21	.22	.39	.09	.16	.19	.21	.38
Mandarin prosody										
Anger	.41	.52	.51	.52	.61	.47	.55	.57	.57	.71
Fear	.25	.37	.38	.39	.60	.23	.33	.37	.35	.58
Happiness	.11	.13	.15	.26	.61	.11	.08	.10	.14	.31
Sadness	.51	.53	.56	.55	.69	.36	.52	.54	.60	.63
Arabic prosody										
Anger	.06	.22	.21	.22	.40	.14	.29	.32	.32	.52
Fear	.17	.21	.29	.23	.40	.20	.28	.27	.30	.58
Happiness	.06	.15	.18	.16	.45	.11	.26	.27	.29	.71
Sadness	.17	.16	.16	.23	.40	.22	.25	.21	.25	.44

**Table 3:** Recognition Latency – Frequency (+ cumulative percent) of stimuli recognized at each gate which contributed to the calculation of Emotion Identification Points (EIP), the frequency of errors (i.e., the target was not identified by GFull), and the mean EIPs in milliseconds (+ standard deviation).

	Chinese group (*n* = 25)							Arab group (*n* = 25)						
Emotion/ Gate	G200	G400	G500	G600	GFull	Errors	EIP Mean (sd)	G200	G400	G500	G600	GFull	Errors	EIP Mean (sd)
Vocalization														
Anger	151 (60%)	15 (66%)	7 (69%)	9 (73%)	19 (80%)	49/250 (20%)	**332ms** (303)	189 (76%)	21 (84%)	17 (91%)	3 (92%)	1 (92%)	19/250 (8%)	**253ms** (146)
Fear	89 (36%)	47 (55%)	17 (62%)	15 (68%)	23 (76%)	59/250 (24%)	**401ms** (278)	102 (41%)	48 (60%)	20 (68%)	10 (72%)	23 (81%)	47/250 (19%)	**386ms** (277)
Happiness-amusement	84 (34%)	79 (66%)	29 (78%)	22 (86%)	18 (93%)	18/250 (7%)	**473ms** (432)	132 (53%)	72 (82%)	16 (88%)	8 (91%)	13 (96%)	9/250 (4%)	**384ms** (385)
Happiness-pleasure	14 (6%)	12 (11%)	15 (17%)	33 (30%)	54 (51%)	122/250 (49%)	**970ms** (608)	26 (10%)	30 (22%)	20 (30%)	25 (40%)	51 (61%)	98/250 (39%)	**837ms** (615)
Sadness	141 (56%)	34 (70%)	16 (76%)	12 (81%)	24 (91%)	23/250 (9%)	**446ms** (502)	113 (45%)	48 (64%)	22 (73%)	17 (80%)	28 (91%)	22/250 (9%)	**490ms** (498)
English prosody														
Anger	39 (13%)	47 (29%)	37 (41%)	35 (53%)	53 (70%)	89/300 (30%)	**840ms** (774)	85 (28%)	65 (50%)	44 (65%)	37 (77%)	28 (86%)	41/300 (14%)	**564ms** (565)
Fear	1 (0%)	7 (2%)	9 (5%)	21 (12%)	60 (33%)	202/300 (67%)	**974ms** (384.15)	15 (5%)	23 (13%)	21 (20%)	46 (35%)	97 (67%)	98/300 (33%)	**865ms** (434)
Happiness	4 (1%)	15 (6%)	10 (10%)	25 (18%)	90 (48%)	156/300 (52%)	**1223ms** (588)	9 (3%)	38 (16%)	20 (22%)	19 (29%)	123 (70%)	91/300 (30%)	**1171ms** (629)
Sadness	35 (12%)	39 (25%)	21 (32%)	38 (44%)	84 (72%)	83/300 (28%)	**1010ms** (758)	41 (14%)	41 (27%)	21 (34%)	21 (41%)	66 (63%)	110/300 (37%)	**949ms** (791)
Mandarin prosody														
Anger	128 (43%)	48 (59%)	16 (64%)	13 (68%)	23 (76%)	72/300 (24%)	**383ms** (293)	175 (58%)	60 (78%)	25 (87%)	15 (92%)	11 (95%)	14/300 (5%)	**334ms** (246)
Fear	67 (22%)	46 (38%)	25 (46%)	28 (55%)	67 (78%)	67/300 (22%)	**716ms** (576)	92 (31%)	48 (47%)	27 (56%)	19 (62%)	52 (79%)	62/300 (21%)	**584ms** (497)
Happiness	19 (6%)	14 (11%)	24 (19%)	44 (34%)	131 (78%)	68/300 (22%)	**1159ms** (677)	5 (2%)	3 (3%)	5 (4%)	14 (9%)	69 (32%)	204/300 (68%)	**1235ms** (525)
Sadness	164 (55%)	29 (64%)	27 (73%)	15 (78%)	32 (89%)	33/300 (11%)	**467ms** (544)	127 (42%)	56 (61%)	21 (68%)	25 (77%)	28 (86%)	43/300 (14%)	**483ms** (508)
Arabic prosody														
Anger	14 (5%)	39 (18%)	11 (21%)	17 (27%)	59 (47%)	160/300 (53%)	**789ms** (455)	27 (9%)	58 (28%)	25 (37%)	21 (44%)	64 (65%)	105/300 (35%)	**702ms** (438)
Fear	13 (4%)	16 (10%)	14 (14%)	15 (19%)	77 (45%)	165/300 (55%)	**849ms** (400)	27 (9%)	22 (16%)	13 (21%)	31 (31%)	99 (64%)	108/300 (36%)	**822ms** (423)
Happiness	7 (2%)	24 (10%)	17 (16%)	26 (25%)	92 (55%)	134/300 (45%)	**1030ms** (514)	26 (9%)	31 (19%)	29 (29%)	30 (39%)	132 (83%)	52/300 (17%)	**990ms** (540)
Sadness	20 (7%)	10 (10%)	21 (17%)	34 (28%)	82 (56%)	133/300 (44%)	**965ms** (557)	31 (10%)	22 (18%)	14 (22%)	28 (32%)	75 (57%)	130/300 (43%)	**878ms** (564)

EIPs considered a total of 250 observations/emotion (25 participants x 10 items) for vocalizations and 300 observations/emotion (25 participants x 12 items) for each prosody condition per group.

Results were broken down as follows: we first characterized how accurately each group recognized specific emotions over time (i.e., as stimuli incrementally increased in duration from gate-to-gate), independently for vocalizations and when listeners judged emotional prosody in their native language. At the same time, we considered the latency associated with stable recognition of emotional targets for each event type, as inferred from the EIPs. These analyses allow comparisons with existing literature that describe the nature and time course of emotion recognition when vocalizations and speech prosody were studied separately. At a second stage, we directly tested whether listeners in our two groups displayed an overall *advantage* in accuracy and/or speed for vocalizations over native prosody, collapsing across emotion types to highlight the broad trends. At a final stage, we focused strictly on how emotions are recognized from prosodic stimuli to evaluate whether linguistic *familiarity* influences recognition of speech-embedded emotions in each group, comparing the performance measures in participants’ native language, second language (L2-English), and a foreign language (again collapsed across emotion types). Full statistical details of the LMMs and post hoc tests conducted on all significant main and interactive effects are reported in S3–S6 Tables. For expository purposes, only the *F*-statistics for significant effects are reported in the text.

### How does emotion recognition unfold from vocalizations?

#### Accuracy.

**Fig 2:**
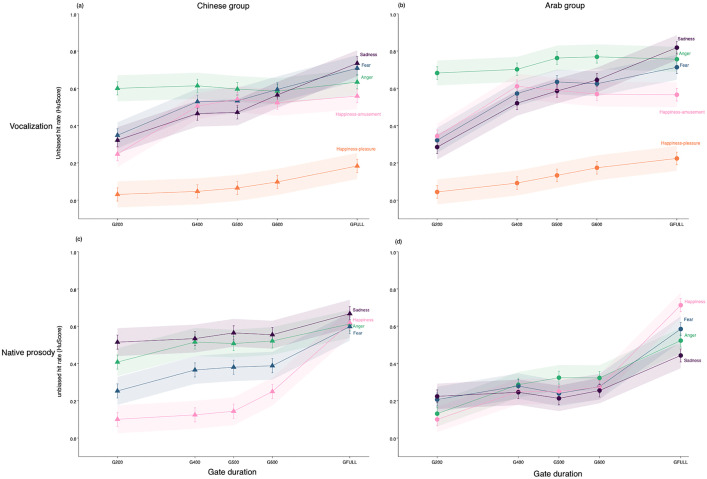
Emotion recognition accuracy (Hu scores) as a function of perceiver group, event type, and stimulus duration. Top panel: Recognition of gated vocalizations by the **A)** Chinese and **B)** Arab participants. Bottom panel: Recognition of gated native speech prosody by the **C)** Chinese and **D)** Arab participants.

Separate group analysis first looked at recognition accuracy for vocalizations (LMM: HuScore (Vocalization) ~ Emotion + Gate + Emotion * Gate + (1| Subject)). Accuracy of each group depended on Emotion type (Arab: *F* = 287.81; *df* = 4, *p* < 0.001; Chinese: *F* = 275. 61, *df* = 4, *p* < 0.001), Gate duration (Arab: *F* = 64.46, *df* = 4, *p* < 0.001; Chinese: *F* = 50.52, *df* = 4, *p* < 0.001), and Emotion x Gate duration (Arab: *F =* 6.43, *df* = 16, *p* < 0.001; Chinese: *F* = 5.78, *df* = 16, *p* < 0.001; S3A Table). Fig 2A,2B shows that while emotional vocalizations had distinct recognition trajectories over time, the accuracy of Chinese and Arab listeners was similar in qualitative and quantitative terms. When accuracy at successive gate durations was compared, data show that anger (growls or shouts) was recognized at high levels based on 200ms of acoustic information (G200) and improved minimally as exposure increased. Happiness-amusement improved when stimulus duration increased from 200–400ms (*p*s < .001), without further improvements between gates to the full stimulus. Fear (screams) and sadness (sobs) also improved significantly between 200–400ms, and then again between G400 to the end of the stimulus (all *p*s* *< .001), suggesting a more incremental buildup of recognition for these signals. Happiness-pleasure was identified very poorly by both groups, and while accuracy improved between 200ms and the end of the utterance (*p*s < .002), recognition remained below chance performance levels for this emotion even when listeners heard the full stimulus.

Limiting stimuli to 200ms yielded superior recognition of anger versus all other vocalizations. Based on 400ms excerpts, anger, fear, and happiness-amusement were recognized at roughly similar rates by each group, exceeding sadness and happiness-pleasure. Virtually no changes occurred in the 400–500ms time range for either group. When stimuli lasted 600ms, only happiness-pleasure was recognized at inferior levels (Arab participants displayed a somewhat prolonged “anger” detection advantage over some vocalizations in the 200–600ms time range, Fig 2B). Recognition of ungated vocalizations (GFull) was highest for anger, fear, and sadness, followed by happiness-amusement, with markedly inferior detection of happiness-pleasure. The fact that pleasure/contentment sounds, in contrast to laughter, were not reliably identified as “happiness” by either group irrespective of exposure time suggests an artefact of our forced-choice design for this category (S1 Table shows that these expressions were labelled as “neutral” approximately half of the time). For this reason, happiness-pleasure was not considered in further analyses; “happiness” in all subsequent models referred solely to amusement/laughter sounds.

#### Latency.

**Fig 3:**
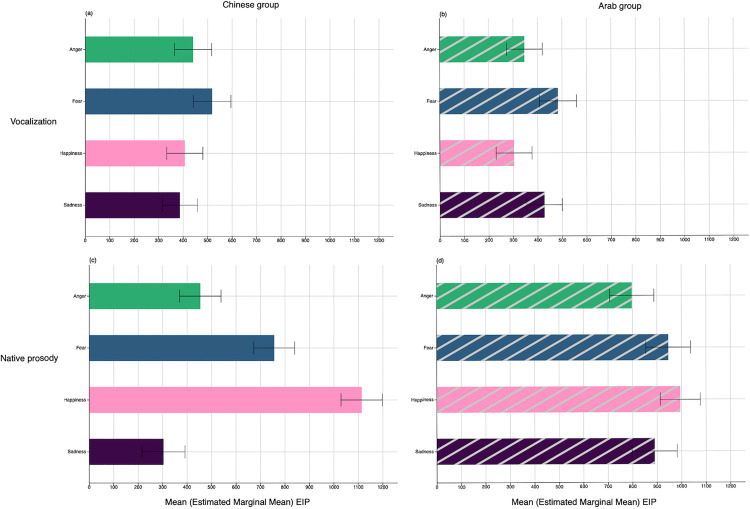
Recognition latency (Emotion Identification Points in milliseconds) as a function of perceiver group and event type. Top panel: Recognition of vocalizations by the **A)** Chinese and **B)** Arab participants. Bottom panel: Recognition of native speech prosody by the **C)** Chinese and **D)** Arab participants.

Analysis of EIPs (omitting happiness-pleasure) considered the time needed to form a stable representation of the four vocalizations between groups, controlling for differences in individual event duration (LMM: EIPtime (Vocalization) ~ Group + Emotion + Group* Emotion + GFullDuration + (1 | Subject))*;* S3B Table). EIPs varied by Emotion (*F* = 9.16, *df* = 3, *p* < 0.001) and Group x Emotion (*F* = 4.04, df = 3, *p* = 0.007). Fig 3A,3B shows that all vocalizations were reliably isolated by listeners in both groups within a narrow ~300–500ms time window. Overall, fear (*M* = 500ms) required significantly more acoustic exposure to isolate than happiness-amusement (*M* = 355ms, *p < *001), anger (*M* = 394ms, *p* < .001), and sadness (*M* = 407ms, *p* = .019). The interaction was explained by small group differences in the significance of emotion-specific contrasts, and evidence that Arab listeners required less time to detect happiness (laughter) than the Chinese (*p = *.041). There was no main effect of perceiver Group on recognition latencies for vocalizations (p = .25, *ns*).

### How does emotion recognition unfold from native speech prosody?

#### Accuracy.

Separate group analyses then considered the buildup of emotion representations when participants heard their native prosody (Chinese = Mandarin, Arab = Arabic; LMM: HuScore (Native prosody) ~ Emotion + Gate + Emotion*Gate + (1 | Subject)). Accuracy differed by Emotion (Arab: *F* = 2.04, *df* = 3, *p* = 0.04; Chinese: *F* = 96.41, *df* = 3, *p* < 0.001), Gate duration (Arab: *F* = 89.04, *df* = 4, *p* < 0.001; Chinese: *F* = 51.08, *df* = 4, *p* < 0.001) and Emotion x Gate duration (Arab: *F* = 4.33, *df* = 12, *p* < 0.001; Chinese: *F* = 5.68, *df* = 12, *p* < 0.001; S4A Table). Native prosody recognition improved incrementally as acoustic details accumulated but more gradually than for vocalizations, with significant improvements usually occurring at longer stimulus exposures (> 600ms, Fig 2C,2D). Moreover, the time course for recognizing specific emotions showed notable variability when Chinese vs. Arab listeners attended to acoustic features in their native language.

For the Chinese group, no significant gate-to-gate improvements occurred for any emotion until >600ms of the utterance was heard; between G600-GFull, recognition then increased significantly for certain emotions (fear, happiness, *p*s < .001). Chinese participants displayed superior recognition of sadness and anger from 200ms prosodic excerpts (anger = sadness > fear > happiness), a pattern that persisted up to 600ms. When Chinese listeners heard ungated utterances (GFull), all emotions were recognized at similar accuracy levels (Fig 2C). For the Arab group, recognition of certain emotions (anger, happiness) improved when prosody increased from 200–400ms; otherwise, no significant gate-to-gate improvements were again observed until listeners heard >600ms of the utterance (G600-GFull), which was characterized by a sharp increase to detect *all* emotions (Fig 2D). Contrary to Chinese listeners, Arab listeners displayed comparable accuracy for the four emotions at short stimulus exposures (G200-G600, except a slight “sadness” advantage at 200ms). Presentation of the full utterance produced marked improvements in recognition of Arabic prosody and differentiation of emotional meanings (happiness > anger = fear > sadness).

While these results show that prosodic representations build up gradually in each language, and target hit rates did not tend to change when exposure times differed minimally (i.e., differences of 100ms or 200ms), accuracy *did* improve significantly within each group when broader time windows were considered (e.g., when comparing G200-G600 or G400-GFull, see post hoc contrasts in S4A Table). Thus, it can be said that prolonged analysis windows at later timepoints seemed to provide relevant acoustic details to recognize emotions from native prosody, in contrast to vocalizations which were isolated within a narrow time range early in the stimulus.

#### Latency.

Direct group comparison of the EIPs for native prosody was then undertaken (LMM: EIPtime (Native prosody) ~ Group + Emotion + Group* Emotion + GFullDuration + (1 | Subject))*;* S4B Table). Recognition latencies depended on Group (*F* = 29.83, *df* = 1, *p* < .001), Emotion (*F* = 83.67, *df* = 3, *p* < .001), and Group x Emotion (*F* = 38.96, *df* = 3, *p* < .001). The time needed to isolate emotions from native prosody in the two languages spanned a sizable range (~300–1100ms), averaging 600–1000ms of stimulus exposure. Overall, sadness (*M* = 597ms) and anger (*M* = 626ms) were recognized faster than fear (*M* = 852ms) and happiness (*M* = 1054ms). These patterns varied somewhat in each perceiver group (Fig 3C,3D), although happiness invariably required the most time to isolate (>1000ms). Anger, sadness, and fear were all recognized from significantly shorter excerpts in Mandarin than in Arabic (all *p*’s < .003), whereas happiness was recognized from shorter stimuli in Arabic vs. Mandarin (*p* = .049).

### Do listeners recognize emotions better from vocalizations than their native language?

Analyses then broadly examined whether vocalizations promoted a recognition advantage in accuracy or speed over native prosody through direct group comparisons (LMM for Accuracy: HuScore (GFull) ~ Group + EventType + Group*EventType + (1 | Subject)+ (1 | Emotion); LMM for Latency: EIPtime ~ Group+ EventType + Group*EventType + GFullDuration + (1 | Subject)+ (1 | Emotion); S5A Table). The model performed on accuracy considered Hu scores at a single gate, GFull, when listeners had all acoustic information available to promote recognition of emotional targets.

#### Accuracy.

**Fig 4:**
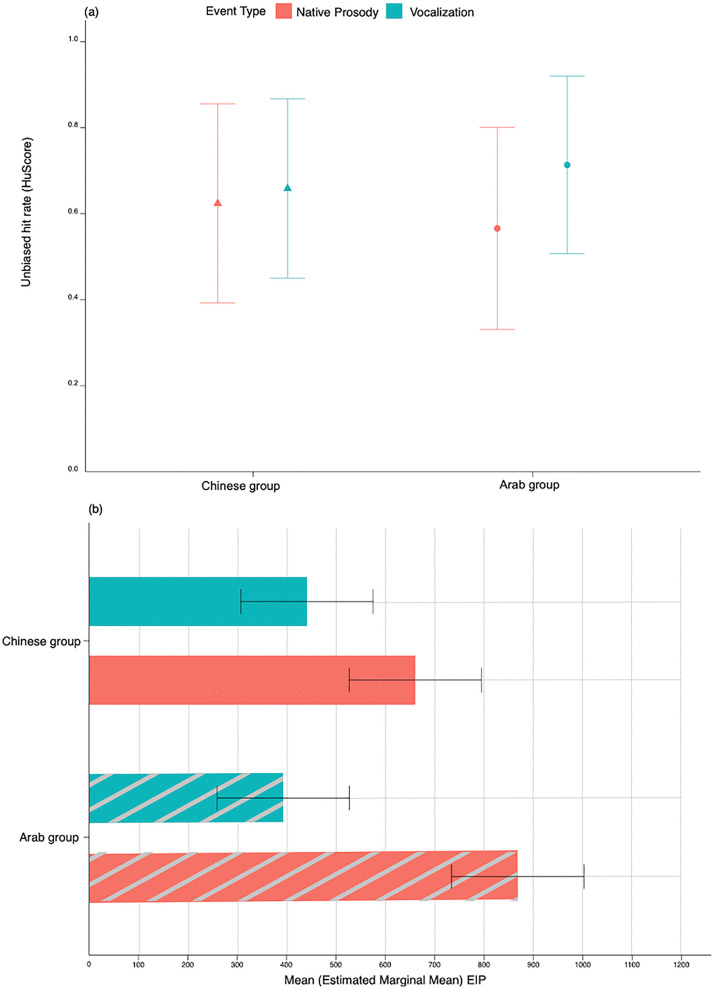
Recognition of vocalizations vs. native prosody by Chinese and Arab participants across emotion types. **A)** Accuracy (Hu score) of each group when ungated expressions were presented (GFull condition). **B)** Corresponding Emotion Identification Points (in milliseconds) by group and event type.

Performance differed significantly by Event type (*F* = 25.95, *df* = 1, *p* < 0.001), pointing to superior accuracy to detect emotional vocalizations (*M* = 0.69) than native prosody (*M* = 0.59) overall. A Group x Event type interaction (*F* = 9.90, *df* = 1, *p* < 0.001) qualified that while the group trends were similar (vocalization > native prosody), this pattern was significant for the Arab listeners (*p* < .001) but not the Chinese (*p* = .169, Fig 4A). When listeners heard expressions in their entirety, there were no Group differences in the ability to recognize emotions in either the vocalization or the native prosody condition *(p*s > .191).

#### Latency.

The time needed to achieve stable recognition of emotions from vocalizations vs. native prosody varied by Group (*F* = 4.90, *df* = 1, *p* = 0.03), vocal Event type (*F* = 489.77, *df* = 1. *p* < 0.001), and their interaction (*F* = 65.66, *df* = 1, *p* < 0.001, S5B Table). Overall, participants recognized vocalizations (*M* = 417ms) from significantly shorter acoustic excerpts than the same emotions expressed through native prosody (*M* = 765ms, *p*s < .001). Vocalizations were recognized at similar latencies by each group (*p* = .222), whereas the time course for recognizing native emotional prosody was language-dependent: here, emotions could be identified from significantly shorter excerpts in Mandarin (Chinese listeners) than in Arabic (Arab listeners, *p < *.001, Fig 4B).

### Does linguistic experience influence emotional prosody recognition?

At a final step, separate group analyses considered patterns of emotion recognition in the three speech prosody conditions defined by the familiarity of each language to each listener group (native, L2-English, foreign), collapsed across emotion types (LMM: HuScore ~ Familiarity + Gate + Familiarity*Gate + (1 | Subject) + (1 | Emotion), S6A Table). These models will reveal if representations underlying emotional prosody are facilitated by (native and/or second) language experience and *when* this knowledge comes into play during event processing [36].

#### Accuracy.

**Fig 5:**
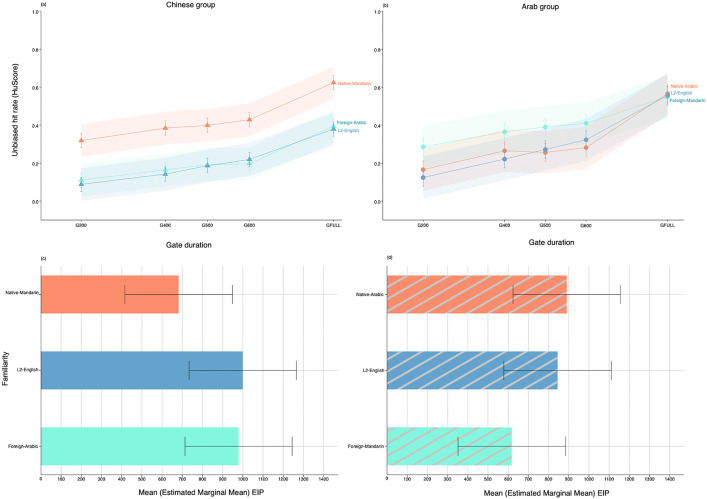
Effects of language familiarity on emotional prosody recognition. Top panel: Accuracy (Hu score) of the **A)** Chinese and **B)** Arab participants over time to recognize prosody in their native language, English as a second language (L2), and in a foreign language. Bottom panel: Corresponding Emotion Identification Points (in milliseconds) for the **C)** Chinese and **D)** Arab participants by language familiarity.

For Chinese listeners, accuracy depended on Gate duration (*F* = 147.3, *df* = 4, *p* < 0.001) and language Familiarity (*F* = 333.27, *df* = 2, *p* < 0.001) in the absence of an interaction. Emotional prosody recognition improved significantly within two principal time windows: between 200–400ms and when listeners had access to all acoustic information in the utterance (G600-GFull); these effects were observed consistently across speech contexts (native, L2, foreign) and were mirrored by the Arab group. Irrespective of stimulus exposure, Chinese listeners identified emotions more accurately from native prosody (Mandarin) than L2-English or foreign prosody (Arabic), exemplifying an ‘ingroup’ recognition advantage for native over non-native forms of prosody [7]. Accuracy in the L2-English vs. foreign language conditions did not differ at any time point (Fig 5A). Arab listeners displayed a unique pattern: accuracy depended on Gate duration (*F* = 186.25, *df* = 4, *p* < 0.001), language Familiarity (*F* = 52.77, *df* = 2, *p* < 0.001), and their interaction (*F* = 5.42, *df* = 8, *p* < 0.001). When prosodic stimuli were short (200–600ms), Arab listeners isolated emotions better in the *foreign* language, Mandarin, than from native (Arabic) or L2-English prosody, which did not differ. When the full utterance became available, Arab listeners showed marked improvements in the native and L2-English conditions, resulting in similar accuracy levels in the three speech prosody contexts (Fig 5B).

To further contextualize these findings, we directly compared Group accuracy at GFull after recoding the speech prosody conditions according to the language of expression (Arabic, English, Mandarin) irrespective of its relevance/familiarity to a particular perceiver group (LMM: HuScore (GFull) ~ Language + Group + Language*Group + (1 | Subject) + (1 | Emotion). Recognition of full prosodic expressions did not differ overall by Language, but differed by Group (*F* = 5.59, *df* = 1, *p* = 0.02) and Group x Language (*F* = 28.73, *df* = 2, *p* < 0.001). Chinese listeners displayed significantly better recognition of Mandarin emotions from full utterances (Mandarin > English = Arabic), whereas Arab listeners displayed no advantage across languages for full utterances (Mandarin = English = Arabic). Emotional prosody in Arabic and English were recognized more accurately by Arab vs. Chinese listeners, whereas the opposite was true for Mandarin (Chinese > Arab, S6B Table).

#### Latency.

Group comparison of EIPs in the three prosody conditions (LMM: EIPtime ~ Group + Familiarity + Group*Familiarity + GFullDuration + (1 | Subject) + (1 | Emotion) uncovered effects of Group (*F* = 9.56, *df* = 1, *p* = 0.003), language Familiarity (*F* = 24.71, *df* = 2, p < 0.001), and Group x Familiarity (*F* = 103.29, *df* = 2, *p* < 0.001, S6C Table). Chinese listeners had significantly shorter recognition latencies for native prosody (*M* = 682ms) than foreign-Arabic (*M* = 979ms) and L2-English prosody (*M* = 999ms, *p*s < .001). In contrast, Arab listeners demonstrated earlier recognition points for foreign-Mandarin prosody (*M* = 618ms) than native-Arabic (*M* = 890ms) and L2-English (*M* = 845ms, *p*s < .001). This contrasting pattern is illustrated in Fig 5C,5D, which shows that emotion representations always stabilized more quickly when listening to speakers of Mandarin irrespective of familiarity. As noted earlier, Chinese listeners displayed faster recognition of their native prosody (*p* < .001), whereas Arab listeners had shorter EIPs than Chinese listeners when judging foreign and L2-English prosody (*p’s* < .001).

## Discussion

Research suggests that in the auditory modality, vocalizations—i.e., grunts, sighs, laughs, sobs, and other brief non-linguistic sounds—signal emotions more rapidly and efficiently than speech prosody [2,5,6]. As phylogenetically older and more reflexive signals [26], spontaneous vocalizations correlate with autonomic and physiological changes in the speaker’s internal state and are produced with less cognitive control and with less acoustic constraints than speech [5,23,24,58]. These factors seem to boost the attentional salience of nonverbal expressions [3,59] and promote the perceptual “clarity” of discrete emotions encoded by nonverbal signals over speech-embedded emotion expressions [2,4,12]. However, there is still a paucity of work that directly compares recognition of the two event types simultaneously.

### Vocalizations promote faster emotion recognition

Our data provide within-subjects verification that listeners tend to be more accurate, but more notably, require substantially *less time* to detect basic emotions communicated by vocalizations than prosodic features of their native language. Listeners required approximately half the exposure time on average to form a stable impression of the speaker’s emotion from vocalizations (Mean = 417ms) than from native prosody (Mean = 765ms). Thus, it can be said that listeners in our study achieved an accurate sense of the intended meaning conveyed by vocalizations more *efficiently*, i.e., in a much shorter time period, than for speech prosody. In evolutionary terms, it is thought that vocalizations were functionally purposed to communicate rapid, ‘honest’ details to conspecifics about emotional events necessary for survival which require minimal conceptual elaboration [18,28]. Our findings similarly suggest that the primary communicative function and main ecological benefit of vocalizations is to transmit acoustic information that allows listeners to construct a categorical representation of the speaker’s emotion state in an *expedited* manner [23,60,61]. Reducing the latency of emotion recognition processes would accelerate adaptive action tendencies associated with discrete emotional signals [1], especially for long-range calls when visual cues about the behavioral context are obscured [62], allowing earlier predictions and reactions in the emotional situation [5].

Anger vocalizations (e.g., shouts) displayed an early detectability advantage and were almost fully recognized from 200ms bursts [45], extending claims that these vocalizations “maximize” the perceived strength and formidability of a speaker when compared to angry speech [6,63]. Increasing stimulus exposure to 400ms resulted in near peak accuracy for all vocalization types with the exception of sadness/crying (which improved somewhat beyond 400ms). These patterns underscore that structural differences within the first 200–400ms of vocalizations (beginning <200ms) provide adequate perceptual information to discriminate and form abstract categorical representations of the emotion qualities communicated by nonverbal acoustic signals [60,61]. Although conducting acoustic analyses was beyond the scope of our study, vocalizations are known to exhibit more nonlinear (aperiodic) acoustic features, and exploit a restricted portion of the acoustic space associated with “roughness” perception that is not used by speech [5,6,18], which could partly explain why these signals are recognized so rapidly. Calculation of EIPs confirmed that the functional significance of all vocalizations was firmly established within a narrow ~300–500ms time window [43] for both Chinese and Arab listeners, who displayed minimal performance differences—despite the fact that vocalizations were extracted from recording databases produced by speakers of various European languages. Arab/Chinese listeners only differed in their recognition latencies for happiness/laughter, although isolation points for this emotion were still rapid (300–400ms). These data bolster claims that nonverbal vocalizations possess robust ‘universal’ acoustic elements [64] that reveal their functional significance to perceivers 300–500ms post-onset of the vocalization. This process can be achieved without prior experience and shows little cultural variation, except possibly for positive emotions [11,12].

### Effects of language on emotional prosody recognition

Emotional prosody was marked by group differences in accuracy, timing, and unique recognition trajectories in each native language, pointing to a more pronounced impact of linguistic and socio-cultural variables on how emotional representations are constructed in speech. When speech excerpts were limited (200–600ms), Chinese listeners displayed superior detection of anger and sadness from native prosody, while Arab listeners displayed a slight advantage for sadness [15,36,41]. The Arab group was notably less accurate to recognize emotions from native prosody when stimulus exposure was short (<600ms). Prosody recognition significantly increased and stabilized only when listeners were fully exposed to native pseudo-utterances (G600-GFull), yielding moderately high emotion hit rates in each group that are comparable to the literature (~ 3x chance accuracy level, e.g., [10,13]). Chinese listeners recognized all emotions with similar accuracy when all acoustic details were available, whereas Arab listeners were more accurate for certain emotions (happiness > anger = fear > sadness).

The timing (EIP) data show that emotions were isolated from prosody at vastly different rates, ranging from 300–1100ms in Mandarin compared to 800–1000ms in Arabic (EIPs in each language were always shortest for sadness and anger and longest for happiness, cf. [36,39,40]). An important conclusion that can be drawn from these data is that emotional prosody recognition is interdependent on language context and structure [13], as suggested by emotion dialect theory [7,46]. Results also show that refining impressions of a speaker’s emotional state from speech often benefits from phrase-final acoustic information [40–42]. In contrast, we found little evidence that event details beyond 600ms post-onset of emotional targets aided recognition of vocalizations for our paradigm.

Our data highlight two critical time periods which may be crucial for extracting details about vocal emotion expressions following event onset: an early window (~0–400ms), which promotes full recognition of nonverbal vocalizations and appears to allow rough differentiation of emotionality/highly salient emotional qualities communicated by speech prosody [3,21,31,43]; and a late extended window (400–1000ms+), which is necessary to monitor and consolidate cues that encode the emotional meaning of prosodic expressions as speech unfolds [36,38,39,41]. The late integration window also serves to incorporate semantic information relevant to the speaker’s emotion state [65,66] and considers phrase-final acoustic cues which impact on how emotional prosody is contextually interpreted [10,41,42]. This processing scheme ensures rapid bottom-up detection of the categorical relevance of motivationally salient vocal signals from acoustic-perceptual information in the early processing interval (including trait impressions of dominance, attractiveness, etc. [67]). At the same time, it explains the *gradual* emergence of stable prosodic representations in speech which tended to build up over longer sampling periods in our data. On average, the EIP data show that the emotional significance of speech prosody—at least for basic negative emotions such as anger, fear and sadness—is reliably established ~500–800ms post-onset of an utterance [36,38–40]. However, unlike vocalizations, these estimates vary considerably across items and languages and can be substantially longer when more socially-constructed emotions, including happiness, are studied (EIPs > 1–2 seconds) [39,41].

Theories of speech perception have proposed that auditory-perceptual integration of basic linguistic units (segments vs. syllables) is accomplished by distinct brain mechanisms that sample acoustic information over different time scales (25–80ms windows for segments, 150–300ms windows for syllables [68]). Along these lines, different forms of vocal signals (vocalizations, prosody) may reveal emotional meanings at unique timepoints owing to distinctly adapted procedures for sampling emotionally relevant acoustic variation over different time scales. Arguably, recognizing emotions in speech depends on a broader analysis window that roughly aligns with major perceptual units that promote linguistic comprehension, such as syllabic units [8,39]. As listeners appraise emotional speech cues, they must also distribute perceptual and cognitive resources to extract linguistic meaning from the acoustic signal; the conscious nature and complexity of this process may contribute to why listeners build more gradual representations of the speaker’s emotion state from prosody when compared to vocalizations.

Vocal communication serves critical functions that regulate human social dynamics, affecting group synchronization, cohesion and how social networks are formed and maintained [62]. Although vocalizations may act as effective long-ranging signals that propagate over distance and are decoded rapidly [5,62], emotional speech tends to serve more “short-ranging” functions selected to modulate within-group behavior and enhance individuating information [69,70]. Thus, humans undoubtedly learn that the *antecedents* of emotional events that are typically expressed in fluent speech are rarely as urgent as those signalled by vocalizations (see [20] for a discussion). Also, experience would reveal that it is more common for people to intentionally manipulate and misrepresent emotional cues to serve their own interpersonal goals [28,71]. Acquired knowledge of these form-function relationships could explain the more variable and sustained analysis period associated with emotional prosody recognition compared to vocalizations, as it is believed that the human capacity to communicate using language, and to communicate *emotions in language,* co-evolved [72].

### On the ingroup advantage for emotional prosody recognition

Several variables are likely to shape how speech-embedded emotions, as well as volitional productions of vocalizations such as laughter [73,74], are assigned value over time and “recognized” in daily interactions; these include cultural preferences/display rules and inferences about the strategic “pragmatic” goals of the speaker in the context of emotional communication [28,75]. Of main interest here, there is mounting evidence that language experience familiarizes people with emotional dialects or vocal expressive “styles” used by particular groups [46], allowing more precise and more rapid construction of emotional prosody representations in listeners’ native vs. a foreign language (ingroup advantage [7]). Our study provides a unique view on this issue, as we employed a fully crossed presentation design involving participants who had no familiarity with the foreign language (Mandarin vs. Arabic) but who all had shared proficiency in English as a second language acquired from childhood, allowing graded effects of familiarity to be evaluated in each group for the first time.

Our findings replicated the ingroup advantage for only one of our two groups: Chinese listeners were more accurate and required less time to name emotions in Mandarin than the other two languages (native > foreign = L2-English, [10,47,76]). This relationship was established after hearing only 200ms speech excerpts and did not temporally evolve (cf. [36]). Presumably, Chinese listeners isolated emotional meanings more efficiently in Mandarin because these stimuli abided by culturally acquired norms of expression familiar to the participants; since performance in L2-English and Arabic (foreign) did not differ, this effect is unlikely to be traced to basic problems in phonological encoding for non-native stimuli.

In contrast, there was no evidence that Arab participants presented an ingroup advantage in accuracy or speed at any stimulus duration (see [77] for similar conclusions using a fully crossed design). Moreover, unlike the Chinese participants, performance of the Arab group evolved as a function of acoustic exposure in each language. When excerpts were brief (200–600ms), Arab listeners were unexpectedly superior in Mandarin for both accuracy and speed (foreign > native = L2-English). However, emotion recognition accuracy for full utterances was comparable in the three languages (Arabic = Mandarin = L2-English). These patterns suggest that Arab listeners relied heavily on utterance-final acoustic cues to form stable impressions of emotion in Arabic and L2-English, and ultimately, they achieved higher recognition rates in these two familiar language contexts than Chinese listeners. However, their *path* to recognition and how they dynamically integrated emotion-related cues in each language was clearly distinct.

Counter-intuitively, our results point out that emotional prosody may be recognized more efficiently at times in a completely unfamiliar language, overriding any advantages conferred by experience with emotional stylings or ‘dialects’ used by native speakers [46]. Although we strived to match our prosodic recordings in the three languages along key dimensions, it seems nonetheless clear that Mandarin speakers supplied representative acoustic cues to emotion that were more perceptually distinct *at earlier stages* in their productions than either the Arabic or English speakers, allowing recognition to stabilize more quickly in this language context for *both* native (Chinese) and foreign (Arab) listeners, irrespective of familiarity. This observation is at odds with claims that emotion recognition abilities are linked to the distance between the expresser and perceiver culture [7]. However, given the sustained time course of emotional prosody analysis, the (bottom-up) perceptual advantage we initially observed for Mandarin prosody disappeared for Arab listeners as acoustic information unfolded over Arabic and English utterances. These patterns remind us that the task of communicating emotions both within and across linguistic boundaries depends not only on top-down factors, i.e., acquired cultural knowledge and contextual expectations about a speaker’s vocal behaviour as described by Dialect Theory [46]—it is fundamentally driven by the *dynamic* quality of the input, i.e., how well speakers execute universally shared ‘affect programmes’ [78] that guide emotional speech recognition at different timepoints, and how well acoustic cues facilitate emotional “inference rules” shared by the speaker-listener [13]. In daily life, speakers encode emotion with different levels of intensity [79,80] and not all individuals are adept at strategically encoding recognizable emotion states in speech [8]. As this research moves forward, the dynamic interplay of stimulus-related features and various knowledge sources that are brought to bear on the act of emotional speech recognition, especially in the case of prosody, will come increasingly to light.

A key novelty of our report was that each perceiver group judged vocalizations and speech prosody in their native, second, and in a foreign language in a fully within-subjects design. While no definitive claims can yet be made, our findings supply no clear evidence that knowing a second language benefited emotional prosody recognition, despite the high English proficiency of our participants. Performance in the L2-English condition mirrored the pattern of foreign prosody (Chinese group) or native prosody (Arab group; cf. [29] for data on English and Hindi). Bhatara et al. [48] reported that L2-English proficiency in a group of French listeners had no effect on prosody recognition (for negative emotions) or seemed to *interfere* in this process (for positive emotions). Other studies suggest that emotional prosody recognition from single words is equally accurate and rapid in one’s native language and L2-English [49]. Our data call for continued monitoring of the relationship between L2 proficiency, emotion recognition, and how acquiring “prosodic-pragmatic competence” in a second language is influenced by different forms of instructional practice [81].

## Conclusions

Our study has several limitations that temper the strength of our claims to some extent. We drew recordings from a variety of sources that involved different speaker characteristics, recording and validation techniques, and which were all produced in controlled laboratory (rather than spontaneous) contexts for expressing emotion. Variability along these dimensions undoubtedly contribute to differences in how distinctively emotions were encoded and therefore perceived across our conditions, impacting our findings in a potentially idiosyncratic manner. In our prosody condition, the extent to which emotion recognition performance observed here in Mandarin-Chinese and Arabic can be generalized to new language contexts is not immediately clear, for example, given the possible relationship between emotional speech and lexical tone recognition in Mandarin (and other tonal languages) [82] and the paucity of previous data on emotion expression in Arabic. Thus, claims about how language familiarity influences emotional prosody should be viewed as suggestive. It should also be noted that auditory gating studies such as the present one can be quite long and seem repetitious to participants, which could affect data quality over time when multiple stimulus types must be evaluated at a large number of gate durations.

Still, our study serves as a useful springboard for new research that compares emotion communication through vocalizations and speech prosody and when both vocal forms are intermixed in natural discourse. Based on our data, it should be emphasized that the recognition of emotions from vocalizations is *not* inherently “better” than for speech prosody, if the quality and quantity of input listeners receive from each form of expression are sufficient. Here, Chinese listeners were just as accurate to identify basic emotions from vocalizations and from native prosody when acoustic exposure to each event was unrestricted; thus, one can say with confidence that a subset of basic emotions can be communicated with equal success (i.e., accuracy or precision) through either nonverbal or linguistic vocal channels. However, emotional meanings were invariably understood much more quickly from nonverbal sounds than prosody [3]. Ensuring that robust representations of a speaker’s emotion state are arrived at *efficiently* may be the core function of vocalizations that distinguishes the two vocal communication subsystems.

## Supporting information

S1 Table Percentage of ‘neutral’ responses assigned by Chinese and Arab participants by vocal event type, emotion, and gate duration. (PDF)

S2 Table Mean confidence ratings (out of 7) for Chinese and Arab participants by vocal event type, emotion, and gate duration. (PDF)

S3 Table Statistical results of models performed on the vocalizations for A) recognition accuracy (Hu scores) and B) recognition latency (Emotion Identification Points). (PDF)

S4 Table Statistical results of models performed on native speech prosody for A) recognition accuracy (Hu scores) and B) recognition latency (Emotion Identification Points). (PDF)

S5 Table Statistical results of models comparing vocalizations and native speech prosody by Group, averaged across emotions, for A) recognition accuracy (Hu scores) and B) recognition latency (Emotion Identification Points). (PDF)

S6 Table Statistical results of models comparing emotional prosody recognition by language for A-B) accuracy (Hu scores) and C) latency (Emotion Identification Points). (PDF)
