# Supplementary material for: Speak or shout? Nonverbal vocalizations promote rapid detection of emotions in vocal communication
Source: PLoS One. 2026 Jan 8;21(1):e0327529. doi: 10.1371/journal.pone.0327529 (PMC12782396; doi:10.1371/journal.pone.0327529)
Supplement: S1 Table — (PDF) [file pone.0327529.s001.pdf]

S1 Table - Percentage of 'neutral' responses assigned by Chinese and Arab participants by vocal event type, emotion, and gate duration.

	Chinese group (*n* = 25)					Arab group (*n* = 25)				
Emotion/ Gate	G200	G400	G500	G600	GFull	G200	G400	G500	G600	GFull
**Vocalization**										
Anger	4%	8%	9%	15%	12%	2%	2%	3%	1%	3%
Fear	17%	9%	8%	8%	8%	12%	2%	5%	3%	3%
Happiness-amusement	22%	14%	6%	9%	1%	12%	3%	5%	1%	0%
Happiness-pleasure	51%	49%	46%	46%	37%	40%	38%	37%	32%	33%
Sadness	4%	3%	5%	5%	1%	3%	2%	1%	1%	0%
**English prosody**										
Anger	41%	38%	28%	27%	18%	32%	22%	20%	11%	7%
Fear	31%	29%	26%	24%	13%	19%	14%	10%	8%	3%
Happiness	37%	35%	38%	32%	34%	24%	24%	20%	19%	22%
Sadness	55%	44%	40%	38%	19%	46%	42%	39%	36%	24%
**Mandarin prosody**										
Anger	1%	9%	8%	9%	8%	0%	2%	2%	3%	2%
Fear	18%	13%	16%	14%	7%	14%	6%	8%	8%	5%
Happiness	17%	16%	22%	19%	8%	6%	7%	10%	9%	13%
Sadness	12%	10%	9%	14%	6%	14%	9%	6%	5%	3%
**Arabic prosody**										
Anger	72%	50%	51%	52%	42%	51%	41%	43%	40%	32%
Fear	40%	39%	32%	36%	25%	21%	28%	33%	32%	14%
Happiness	59%	56%	50%	52%	34%	54%	50%	49%	46%	13%
Sadness	57%	67%	63%	58%	40%	52%	57%	61%	56%	40%
