# Supplementary material for: Speak or shout? Nonverbal vocalizations promote rapid detection of emotions in vocal communication
Source: PLoS One. 2026 Jan 8;21(1):e0327529. doi: 10.1371/journal.pone.0327529 (PMC12782396; doi:10.1371/journal.pone.0327529)
Supplement: S2 Table — (PDF) [file pone.0327529.s002.pdf]

S2 Table - Mean confidence ratings (out of 7) for Chinese and Arab participants by vocal event type, emotion, and gate duration.

	**Chinese group** (*n* = 25)					**Arab group** (*n* = 25)				
**Emotion/Gate**	G200	G400	G500	G600	GFull	G200	G400	G500	G600	GFull
**Vocalization**										
Anger	5.74	5.78	5.98	5.92	6.27	5.79	5.98	6.10	6.22	6.47
Fear	5.27	5.88	6.06	6.12	6.38	5.14	5.83	6.00	6.02	6.35
Happiness-amusement	5.00	5.86	6.21	6.21	6.60	5.20	5.90	6.18	6.34	6.55
Happiness-pleasure	4.74	5.11	5.33	5.58	5.90	4.69	4.82	5.06	5.06	5.73
Sadness	5.48	6.00	6.26	6.22	6.58	5.41	5.93	6.02	6.17	6.51
**English prosody**										
Anger	4.35	4.90	5.21	5.43	6.04	4.56	4.77	5.18	5.50	6.06
Fear	4.51	4.78	5.08	5.18	5.79	4.57	4.76	5.16	5.23	5.84
Happiness	4.49	4.77	5.02	5.12	5.73	4.66	4.79	5.02	5.06	5.64
Sadness	4.35	4.83	5.07	5.06	5.77	4.39	4.65	4.96	5.12	5.60
**Mandarin prosody**										
Anger	5.57	5.75	5.72	5.84	6.08	5.56	5.57	5.70	5.92	6.09
Fear	4.88	5.17	5.38	5.49	5.94	4.79	4.72	5.01	5.06	5.69
Happiness	4.90	5.02	5.33	5.54	6.06	4.89	4.57	4.75	4.88	5.15
Sadness	5.34	5.60	5.68	5.67	6.08	4.99	5.20	5.37	5.52	6.04
**Arabic prosody**										
Anger	4.20	4.68	4.88	4.98	5.48	4.43	4.75	4.94	5.20	5.58
Fear	4.33	4.74	5.01	4.97	5.48	4.61	4.31	4.50	4.91	5.27
Happiness	4.24	4.64	4.75	5.01	5.60	4.87	4.87	5.09	5.06	5.59
Sadness	4.22	4.75	4.73	4.93	5.53	4.78	4.77	4.94	5.10	5.50
