# Supplementary material for: Speak or shout? Nonverbal vocalizations promote rapid detection of emotions in vocal communication
Source: PLoS One. 2026 Jan 8;21(1):e0327529. doi: 10.1371/journal.pone.0327529 (PMC12782396; doi:10.1371/journal.pone.0327529)
Supplement: S3 Table — (PDF) [file pone.0327529.s003.pdf]

S3 Table. Statistical results of models performed on the vocalizations for A) recognition accuracy (Hu scores) and B) recognition latency (Emotion Identification Points).

S3A - Analysis of recognition accuracy (Hu scores) for vocalizations

LMM (*HuScore (Vocalization) ~ Emotion + Gate + Emotion*Gate + (1| Subject))* and post hoc tests showing recognition accuracy for vocalizations by emotion and gate duration, separately for Chinese and Arab participants.

Chinese Group LMM

Predictors	*Estimates*	*CI*	*p*	*df*
(Intercept)	0.60	0.53 – 0.67	**<0.001**	598.00
Emotion [Fear]	-0.25	-0.33 – -0.17	**<0.001**	598.00
Emotion [Happiness_ amusement]	-0.35	-0.43 – -0.27	**<0.001**	598.00
Emotion [Happiness_pleasure]	-0.57	-0.65 – -0.49	**<0.001**	598.00
Emotion [Sadness]	-0.28	-0.36 – -0.20	**<0.001**	598.00
Gate [G400]	0.01	-0.07 – 0.09	0.736	598.00
Gate [G500]	-0.00	-0.08 – 0.08	0.926	598.00
Gate [G600]	-0.02	-0.10 – 0.06	0.705	598.00
Gate [GFULL]	0.03	-0.05 – 0.11	0.410	598.00
Emotion [Fear] * Gate [G400]	0.17	0.05 – 0.28	**0.004**	598.00
Emotion [Happiness_ amusement] * Gate [G400]	0.24	0.13 – 0.36	**<0.001**	598.00

Emotion [Happiness_pleasure] * Gate [G400]	0.00	-0.11 – 0.12	0.965	598.00
Emotion [Sadness] * Gate [G400]	0.13	0.02 – 0.24	**0.025**	598.00
Emotion [Fear] * Gate [G500]	0.19	0.08 – 0.30	**0.001**	598.00
Emotion [Happiness_ amusement] * Gate [G500]	0.30	0.19 – 0.41	**<0.001**	598.00
Emotion [Happiness_pleasure] * Gate [G500]	0.04	-0.07 – 0.15	0.504	598.00
Emotion [Sadness] * Gate [G500]	0.15	0.04 – 0.27	**0.008**	598.00
Emotion [Fear] * Gate [G600]	0.26	0.15 – 0.37	**<0.001**	598.00
Emotion [Happiness_ amusement] * Gate [G600]	0.29	0.18 – 0.40	**<0.001**	598.00
Emotion [Happiness_pleasure] * Gate [G600]	0.08	-0.03 – 0.20	0.152	598.00
Emotion [Sadness] * Gate [G600]	0.26	0.15 – 0.37	**<0.001**	598.00
Emotion [Fear] * Gate [GFULL]	0.33	0.21 – 0.44	**<0.001**	598.00
Emotion [Happiness_ amusement] * Gate [GFULL]	0.28	0.17 – 0.39	**<0.001**	598.00

Emotion [Happiness_pleasure] * Gate [GFULL]	0.12	0.01 – 0.23	**0.039**	598.00
Emotion [Sadness] * Gate [GFULL]	0.38	0.27 – 0.49	**<0.001**	598.00
**Random Effects**				
σ^2	0.02			
$\tau_{00\ Subject}$	0.01			
ICC	0.34			
$N_{Subject}$	25			
Observations	625			
Marginal R^2 / Conditional R^2	0.583 / 0.725			

Post hoc test results for LMM S3A (Chinese group)

contrast	Emotion	estimate	*SE*	*df*	*t*	*p*
G200 - G400	Anger	-0.01	0.04	600.00	-0.34	.997
G200 - G500	Anger	0.00	0.04	600.00	0.09	1.00
G200 - G600	Anger	0.02	0.04	600.00	0.38	.996
G200 - GFULL	Anger	-0.03	0.04	600.00	-0.82	.923
G400 - G500	Anger	0.02	0.04	600.00	0.43	.993

G400 - G600	Anger	0.03	0.04	600.00	0.72	.953
G400 - GFULL	Anger	-0.02	0.04	600.00	-0.49	.989
G500 - G600	Anger	0.01	0.04	600.00	0.28	.999
G500 - GFULL	Anger	-0.04	0.04	600.00	-0.92	.890
G600 - GFULL	Anger	-0.05	0.04	600.00	-1.20	.750
G200 - G400	Fear	-0.18	0.04	600.00	-4.44	< .001***
G200 - G500	Fear	-0.19	0.04	600.00	-4.55	< .001***
G200 - G600	Fear	-0.25	0.04	600.00	-6.05	< .001***
G200 - GFULL	Fear	-0.36	0.04	600.00	-8.87	< .001***
G400 - G500	Fear	-0.00	0.04	600.00	-0.11	1.00
G400 - G600	Fear	-0.07	0.04	600.00	-1.61	.489
G400 - GFULL	Fear	-0.18	0.04	600.00	-4.43	< .001***
G500 - G600	Fear	-0.06	0.04	600.00	-1.50	.561
G500 - GFULL	Fear	-0.18	0.04	600.00	-4.32	< .001***
G600 - GFULL	Fear	-0.11	0.04	600.00	-2.82	.040*
G200 - G400	Happiness_amusement	-0.26	0.04	600.00	-6.33	< .001***
G200 - G500	Happiness_ amusement	-0.30	0.04	600.00	-7.26	< .001***

G200 - G600	Happiness_ amusement	-0.28	0.04600.00	-6.79	< .001***
G200 - GFULL	Happiness_ amusement	-0.31	0.04600.00	-7.67	< .001***
G400 - G500	Happiness_ amusement	-0.04	0.04600.00	-0.92	.887
G400 - G600	Happiness_ amusement	-0.02	0.04600.00	-0.45	.991
G400 - GFULL	Happiness_ amusement	-0.05	0.04600.00	-1.33	.671
G500 - G600	Happiness_ amusement	0.02	0.04600.00	0.47	.990
G500 - GFULL	Happiness_ amusement	-0.02	0.04600.00	-0.41	.994
G600 - GFULL	Happiness_ amusement	-0.04	0.04600.00	-0.88	.904
G200 - G400	Happiness_pleasure	-0.02	0.04600.00	-0.40	.995
G200 - G500	Happiness_pleasure	-0.03	0.04600.00	-0.85	.914
G200 - G600	Happiness_pleasure	-0.07	0.04600.00	-1.65	.467
G200 - GFULL	Happiness_pleasure	-0.15	0.04600.00	-3.75	.002**
G400 - G500	Happiness_pleasure	-0.02	0.04600.00	-0.45	.991
G400 - G600	Happiness_pleasure	-0.05	0.04600.00	-1.25	.722
G400 - GFULL	Happiness_pleasure	-0.14	0.04600.00	-3.35	.008**
G500 - G600	Happiness_pleasure	-0.03	0.04600.00	-0.80	.932
G500 - GFULL	Happiness_pleasure	-0.12	0.04600.00	-2.90	.032*

G600 - GFULL	Happiness_pleasure	-0.09	0.04	600.00	-2.10	.221
G200 - G400	Sadness	-0.14	0.04	600.00	-3.51	.004**
G200 - G500	Sadness	-0.15	0.04	600.00	-3.67	.002**
G200 - G600	Sadness	-0.24	0.04	600.00	-5.97	< .001***
G200 - GFULL	Sadness	-0.41	0.04	600.00	-10.15	< .001***
G400 - G500	Sadness	-0.01	0.04	600.00	-0.16	1.00
G400 - G600	Sadness	-0.10	0.04	600.00	-2.46	.102
G400 - GFULL	Sadness	-0.27	0.04	600.00	-6.64	< .001***
G500 - G600	Sadness	-0.09	0.04	600.00	-2.30	.147
G500 - GFULL	Sadness	-0.26	0.04	600.00	-6.48	< .001***
G600 - GFULL	Sadness	-0.17	0.04	600.00	-4.18	< .001***

Arab Group LMM

Predictors	*Estimates*	*CI*	*p*	*df*
(Intercept)	0.68	0.62 – 0.75	**<0.001**	598.00
Emotion [Fear]	-0.36	-0.44 – -0.28	**<0.001**	598.00
Emotion [Happiness_ amusement]	-0.34	-0.42 – -0.26	**<0.001**	598.00

Emotion [Happiness_pleasure]	-0.64	-0.72 – -0.56	**<0.001**	598.00
Emotion [Sadness]	-0.40	-0.48 – -0.32	**<0.001**	598.00
Gate [G400]	0.02	-0.06 – 0.10	0.637	598.00
Gate [G500]	0.08	-0.00 – 0.16	0.055	598.00
Gate [G600]	0.09	0.00 – 0.17	**0.038**	598.00
Gate [GFULL]	0.07	-0.01 – 0.16	0.077	598.00
Emotion [Fear] * Gate [G400]	0.23	0.12 – 0.35	**<0.001**	598.00
Emotion [Happiness_ amusement] * Gate [G400]	0.25	0.13 – 0.36	**<0.001**	598.00
Emotion [Happiness_pleasure] * Gate [G400]	0.03	-0.09 – 0.14	0.636	598.00
Emotion [Sadness] * Gate [G400]	0.22	0.10 – 0.33	**<0.001**	598.00
Emotion [Fear] * Gate [G500]	0.23	0.12 – 0.35	**<0.001**	598.00
Emotion [Happiness_ amusement] * Gate [G500]	0.17	0.05 – 0.28	**0.005**	598.00
Emotion [Happiness_pleasure] * Gate [G500]	0.01	-0.11 – 0.12	0.887	598.00
Emotion [Sadness] * Gate [G500]	0.22	0.10 – 0.34	**<0.001**	598.00

Emotion [Fear] * Gate [G600]	0.22	0.10 – 0.33	**<0.001**	598.00
Emotion [Happiness_ amusement] * Gate [G600]	0.14	0.02 – 0.25	**0.019**	598.00
Emotion [Happiness_pleasure] * Gate [G600]	0.04	-0.07 – 0.16	0.468	598.00
Emotion [Sadness] * Gate [G600]	0.27	0.16 – 0.39	**<0.001**	598.00
Emotion [Fear] * Gate [GFULL]	0.32	0.20 – 0.43	**<0.001**	598.00
Emotion [Happiness_ amusement] * Gate [GFULL]	0.15	0.03 – 0.26	**0.012**	598.00
Emotion [Happiness_pleasure] * Gate [GFULL]	0.11	-0.01 – 0.22	0.074	598.00
Emotion [Sadness] * Gate [GFULL]	0.46	0.34 – 0.58	**<0.001**	598.00
**Random Effects**				
σ^2	0.02			
$\tau_{00\ Subject}$	0.01			
ICC	0.24			
$N_{Subject}$	25			
Observations	625			
Marginal R^2 / Conditional R^2	0.647 / 0.733			

Post hoc test results for LMM S3A (Arab group)

contrast	Emotion	estimate	*SE*	*df*	*t*	*p*
G200 - G400	Anger	-0.02	0.04	600.00	-0.47	.990
G200 - G500	Anger	-0.08	0.04	600.00	-1.93	.305
G200 - G600	Anger	-0.09	0.04	600.00	-2.08	.229
G200 - GFULL	Anger	-0.07	0.04	600.00	-1.77	.391
G400 - G500	Anger	-0.06	0.04	600.00	-1.45	.593
G400 - G600	Anger	-0.07	0.04	600.00	-1.61	.491
G400 - GFULL	Anger	-0.05	0.04	600.00	-1.30	.691
G500 - G600	Anger	-0.01	0.04	600.00	-0.16	1.00
G500 - GFULL	Anger	0.01	0.04	600.00	0.15	1.00
G600 - GFULL	Anger	0.01	0.04	600.00	0.31	.998
G200 - G400	Fear	-0.25	0.04	600.00	-6.01	< .001***
G200 - G500	Fear	-0.31	0.04	600.00	-7.51	< .001***
G200 - G600	Fear	-0.30	0.04	600.00	-7.27	< .001***
G200 - GFULL	Fear	-0.39	0.04	600.00	-9.39	< .001***

Post hoc test results for LMM S3A (Arab group)

contrast	Emotion	estimate	*SE*	*df*	*t*	*p*
G400 - G500	Fear	-0.06	0.04	600.00	-1.49	.568
G400 - G600	Fear	-0.05	0.04	600.00	-1.26	.717
G400 - GFULL	Fear	-0.14	0.04	600.00	-3.38	.007**
G500 - G600	Fear	0.01	0.04	600.00	0.23	.999
G500 - GFULL	Fear	-0.08	0.04	600.00	-1.89	.326
G600 - GFULL	Fear	-0.09	0.04	600.00	-2.12	.213
G200 - G400	Happiness_ amusement	-0.27	0.04	600.00	-6.41	< .001***
G200 - G500	Happiness_ amusement	-0.25	0.04	600.00	-5.89	< .001***
G200 - G600	Happiness_ amusement	-0.23	0.04	600.00	-5.41	< .001***
G200 - GFULL	Happiness_ amusement	-0.22	0.04	600.00	-5.33	< .001***
G400 - G500	Happiness_ amusement	0.02	0.04	600.00	0.52	.985
G400 - G600	Happiness_ amusement	0.04	0.04	600.00	1.00	.854
G400 - GFULL	Happiness_ amusement	0.05	0.04	600.00	1.08	.815
G500 - G600	Happiness_ amusement	0.02	0.04	600.00	0.48	.989
G500 - GFULL	Happiness_ amusement	0.02	0.04	600.00	0.56	.980

Post hoc test results for LMM S3A (Arab group)

contrast	Emotion	estimate	*SE*	*df*	*t*	*p*
G600 - GFULL	Happiness_ amusement	0.00	0.04	600.00	0.08	1.00
G200 - G400	Happiness_pleasure	-0.05	0.04	600.00	-1.14	.784
G200 - G500	Happiness_pleasure	-0.09	0.04	600.00	-2.13	.210
G200 - G600	Happiness_pleasure	-0.13	0.04	600.00	-3.11	.017*
G200 - GFULL	Happiness_pleasure	-0.18	0.04	600.00	-4.31	< .001***
G400 - G500	Happiness_pleasure	-0.04	0.04	600.00	-0.98	.862
G400 - G600	Happiness_pleasure	-0.08	0.04	600.00	-1.97	.283
G400 - GFULL	Happiness_pleasure	-0.13	0.04	600.00	-3.16	.014*
G500 - G600	Happiness_pleasure	-0.04	0.04	600.00	-0.98	.863
G500 - GFULL	Happiness_pleasure	-0.09	0.04	600.00	-2.18	.189
G600 - GFULL	Happiness_pleasure	-0.05	0.04	600.00	-1.20	.753
G200 - G400	Sadness	-0.24	0.04	600.00	-5.64	< .001***
G200 - G500	Sadness	-0.30	0.04	600.00	-7.21	< .001***
G200 - G600	Sadness	-0.36	0.04	600.00	-8.62	< .001***
G200 - GFULL	Sadness	-0.53	0.04	600.00	-12.78	< .001***

Post hoc test results for LMM S3A (Arab group)

contrast	Emotion	estimate	*SE*	*df*	*t*	*p*
G400 - G500	Sadness	-0.07	0.04	600.00	-1.57	.517
G400 - G600	Sadness	-0.12	0.04	600.00	-2.98	.025*
G400 - GFULL	Sadness	-0.30	0.04	600.00	-7.14	< .001***
G500 - G600	Sadness	-0.06	0.04	600.00	-1.41	.619
G500 - GFULL	Sadness	-0.23	0.04	600.00	-5.57	< .001***
G600 - GFULL	Sadness	-0.17	0.04	600.00	-4.16	< .001***

S3B – Analysis of recognition latency (Emotion Identification Points) for vocalizations

Results of LMM (*EIPtime ~ Group + Emotion + Group*Emotion + GFullDuration + (1 | Subject)*) and post hoc tests showing recognition latencies for vocalizations by Group (Chinese, Arab) and emotion type (anger, fear, happiness-amusement, sadness). Note that "happiness" referred only to items representing the category "happiness-amusement" for this analysis.

EIP as a function of Emotional vocalization and Group

Predictors	*Estimates*	*CI*	*p*	*df*
(Intercept)	25.18	-68.29 – 118.64	0.597	1743.00
Group [Chinese]	93.87	-5.47 – 193.21	0.064	1743.00
Emotion [Fear]	136.43	69.89 – 202.97	**<0.001**	1743.00
Emotion [Happiness]	-43.27	-121.39 – 34.84	0.277	1743.00
Emotion [Sadness]	80.46	4.99 – 155.93	**0.037**	1743.00
FullDuration ms	0.23	0.17 – 0.30	**<0.001**	1743.00
Group [Chinese] × Emotion [Fear]	-58.76	-155.20 – 37.68	0.232	1743.00
Group [Chinese] × Emotion [Happiness]	9.34	-82.58 – 101.25	0.842	1743.00
Group [Chinese] × Emotion [Sadness]	-134.59	-227.44 – -41.74	**0.005**	1743.00
**Random Effects**				
σ^2	123250.92			
$\tau_{00\ Subject}$	17555.42			
ICC	0.12			
$N_{Subject}$	50			

Observations	1754
Marginal R^2 / Conditional R^2	0.062 / 0.179

Post hoc test results of LMM_S3B (divided by Emotion)

contrast	Emotion	estimate	*SE*	*df*	*t*	*p*
Arabic - Chinese	Anger	-93.87	50.65	89.34	-1.85	.067
Arabic - Chinese	Fear	-35.11	51.76	97.04	-0.68	.499
Arabic - Chinese	Happiness	-103.20	49.59	82.09	-2.08	.041*
Arabic - Chinese	Sadness	40.72	49.91	84.61	0.82	.417

Post hoc test results of LMM_S3B (divided by Group)

contrast	Group	estimate	*SE*	*df*	*t*	*p*
Anger - Fear	Arabic	-136.43	33.93	1,697.02	-4.02	< .001***
Anger - Happiness	Arabic	43.27	39.83	1,691.67	1.09	.698
Anger - Sadness	Arabic	-80.46	38.48	1,693.01	-2.09	.156

Post hoc test results of LMM_S3B (divided by Group)

contrast	Group	estimate	*SE*	*df*	*t*	*p*
Fear - Happiness	Arabic	179.70	41.61	1,694.32	4.32	< .001***
Fear - Sadness	Arabic	55.97	40.18	1,693.72	1.39	.504
Happiness - Sadness	Arabic	-123.73	32.62	1,692.14	-3.79	.001***
Anger - Fear	Chinese	-77.68	35.62	1,697.76	-2.18	.129
Anger - Happiness	Chinese	33.93	41.20	1,695.07	0.82	.843
Anger - Sadness	Chinese	54.13	39.79	1,695.57	1.36	.525
Fear - Happiness	Chinese	111.61	42.03	1,692.86	2.66	.040*
Fear - Sadness	Chinese	131.80	40.62	1,696.06	3.24	.007**
Happiness - Sadness	Chinese	20.19	32.97	1,696.49	0.61	.928

(collapsed on Group)

contrast	estimate	*SE*	*df*	*t*	*p*
Anger - Fear	-107.05	24.61	1,697.35	-4.35	< .001***
Anger - Happiness	38.60	33.06	1,693.41	1.17	.647
Anger - Sadness	-13.17	31.17	1,693.90	-0.42	.975
Fear - Happiness	145.66	34.24	1,692.95	4.25	< .001***
Fear - Sadness	93.89	32.32	1,693.80	2.91	.019*
Happiness - Sadness	-51.77	23.29	1,694.41	-2.22	.117
