# Supplementary material for: Speak or shout? Nonverbal vocalizations promote rapid detection of emotions in vocal communication
Source: PLoS One. 2026 Jan 8;21(1):e0327529. doi: 10.1371/journal.pone.0327529 (PMC12782396; doi:10.1371/journal.pone.0327529)
Supplement: S4 Table — (PDF) [file pone.0327529.s004.pdf]

S4 Table. Statistical results of models performed on native speech prosody for A) recognition accuracy (Hu scores) and B) recognition latency (Emotion Identification Points).

S4A – Analysis of recognition accuracy (Hu scores) for native prosody

Linear Mixed Model (*HuScore (Native prosody) ~ Emotion + Gate + Emotion*Gate + (1| Subject))* and post hoc tests showing recognition accuracy for native speech prosody by emotion and gate duration, separately for Chinese and Arab participants.

Chinese Group LMM

Predictors	*Estimates*	*CI*	*p*	*df*
(Intercept)	0.41	0.33 – 0.48	**<0.001**	478.00
Emotion [Fear]	-0.16	-0.24 – -0.07	**0.001**	478.00
Emotion [Happiness]	-0.31	-0.40 – -0.22	**<0.001**	478.00
Emotion [Sadness]	0.11	0.02 – 0.20	**0.019**	478.00
Gate [G400]	0.11	0.02 – 0.20	**0.019**	478.00
Gate [G500]	0.10	0.01 – 0.19	**0.029**	478.00
Gate [G600]	0.11	0.02 – 0.20	**0.013**	478.00
Gate [GFULL]	0.20	0.12 – 0.29	**<0.001**	478.00
Emotion [Fear] * Gate [G400]	0.01	-0.12 – 0.13	0.936	478.00
Emotion [Happiness] * Gate [G400]	-0.08	-0.21 – 0.04	0.194	478.00
Emotion [Sadness] * Gate [G400]	-0.09	-0.22 – 0.04	0.171	478.00

Emotion [Fear] * Gate [G500]	0.03	-0.10 – 0.15	0.663	478.00
Emotion [Happiness] * Gate [G500]	-0.06	-0.18 – 0.07	0.382	478.00
Emotion [Sadness] * Gate [G500]	-0.05	-0.18 – 0.08	0.442	478.00
Emotion [Fear] * Gate [G600]	0.02	-0.10 – 0.15	0.735	478.00
Emotion [Happiness] * Gate [G600]	0.04	-0.09 – 0.16	0.583	478.00
Emotion [Sadness] * Gate [G600]	-0.07	-0.20 – 0.05	0.254	478.00
Emotion [Fear] * Gate [GFULL]	0.14	0.01 – 0.27	**0.029**	478.00
Emotion [Happiness] * Gate [GFULL]	0.31	0.19 – 0.44	**<0.001**	478.00
Emotion [Sadness] * Gate [GFULL]	-0.05	-0.18 – 0.07	0.420	478.00
**Random Effects**				
σ^2	0.03			
$\tau_{00\ Subject}$	0.01			
ICC	0.28			
$N_{Subject}$	25			
Observations	500			
Marginal R^2 / Conditional R^2	0.448 / 0.602			

Post hoc test results of LMM S4A (Chinese group)

contrast	Emotion	estimate	*SE*	*df*	*t*	*p*
G200 - G400	Anger	-0.11	0.05	475.00	-2.36	.128
G200 - G500	Anger	-0.10	0.05	475.00	-2.18	.187
G200 - G600	Anger	-0.11	0.05	475.00	-2.49	.095
G200 - GFULL	Anger	-0.20	0.05	475.00	-4.49	< .001***
G400 - G500	Anger	0.01	0.05	475.00	0.18	1.00
G400 - G600	Anger	-0.01	0.05	475.00	-0.13	1.00
G400 - GFULL	Anger	-0.10	0.05	475.00	-2.13	.208
G500 - G600	Anger	-0.01	0.05	475.00	-0.30	.998
G500 - GFULL	Anger	-0.11	0.05	475.00	-2.31	.144
G600 - GFULL	Anger	-0.09	0.05	475.00	-2.00	.265
G200 - G400	Fear	-0.11	0.05	475.00	-2.48	.098

Post hoc test results of LMM S4A (Chinese group)

contrast	Emotion	estimate	*SE*	*df*	*t*	*p*
G200 - G500	Fear	-0.13	0.05	475.00	-2.80	.042*
G200 - G600	Fear	-0.14	0.05	475.00	-2.97	.026*
G200 - GFULL	Fear	-0.35	0.05	475.00	-7.58	< .001***
G400 - G500	Fear	-0.01	0.05	475.00	-0.32	.998
G400 - G600	Fear	-0.02	0.05	475.00	-0.49	.988
G400 - GFULL	Fear	-0.23	0.05	475.00	-5.11	< .001***
G500 - G600	Fear	-0.01	0.05	475.00	-0.17	1.00
G500 - GFULL	Fear	-0.22	0.05	475.00	-4.78	< .001***
G600 - GFULL	Fear	-0.21	0.05	475.00	-4.61	< .001***
G200 - G400	Happiness	-0.02	0.05	475.00	-0.52	.985
G200 - G500	Happiness	-0.04	0.05	475.00	-0.95	.878
G200 - G600	Happiness	-0.15	0.05	475.00	-3.27	.010*

Post hoc test results of LMM S4A (Chinese group)

contrast	Emotion	estimate	*SE*	*df*	*t*	*p*
G200 - GFULL	Happiness	-0.52	0.05	475.00	-11.36	< .001***
G400 - G500	Happiness	-0.02	0.05	475.00	-0.42	.993
G400 - G600	Happiness	-0.12	0.05	475.00	-2.74	.049*
G400 - GFULL	Happiness	-0.49	0.05	475.00	-10.84	< .001***
G500 - G600	Happiness	-0.11	0.05	475.00	-2.32	.141
G500 - GFULL	Happiness	-0.47	0.05	475.00	-10.42	< .001***
G600 - GFULL	Happiness	-0.37	0.05	475.00	-8.10	< .001***
G200 - G400	Sadness	-0.02	0.05	475.00	-0.42	.993
G200 - G500	Sadness	-0.05	0.05	475.00	-1.10	.809
G200 - G600	Sadness	-0.04	0.05	475.00	-0.87	.906
G200 - GFULL	Sadness	-0.15	0.05	475.00	-3.35	.008**
G400 - G500	Sadness	-0.03	0.05	475.00	-0.67	.962

Post hoc test results of LMM S4A (Chinese group)						
contrast	Emotion	estimate	*SE*	*df*	*t*	*p*
G400 - G600	Sadness	-0.02	0.05	475.00	-0.45	.991
G400 - GFULL	Sadness	-0.13	0.05	475.00	-2.93	.029*
G500 - G600	Sadness	0.01	0.05	475.00	0.22	.999
G500 - GFULL	Sadness	-0.10	0.05	475.00	-2.26	.161
G600 - GFULL	Sadness	-0.11	0.05	475.00	-2.48	.097

Arab Group LMM

Predictors	*Estimates*	*CI*	*p*	*df*
(Intercept)	0.13	0.06 – 0.20	**<0.001**	478.00
Emotion [Fear]	0.08	-0.01 – 0.17	0.096	478.00
Emotion [Happiness]	-0.03	-0.12 – 0.06	0.501	478.00
Emotion [Sadness]	0.09	0.00 – 0.18	**0.042**	478.00
Gate [G400]	0.16	0.07 – 0.24	**0.001**	478.00
Gate [G500]	0.19	0.10 – 0.28	**<0.001**	478.00
Gate [G600]	0.19	0.10 – 0.28	**<0.001**	478.00

Gate [GFULL]	0.39	0.30 – 0.48	**<0.001**	478.00
Emotion [Fear] * Gate [G400]	-0.08	-0.21 – 0.04	0.199	478.00
Emotion [Happiness] * Gate [G400]	-0.01	-0.13 – 0.12	0.922	478.00
Emotion [Sadness] * Gate [G400]	-0.13	-0.26 – -0.01	**0.039**	478.00
Emotion [Fear] * Gate [G500]	-0.16	-0.29 – -0.03	**0.013**	478.00
Emotion [Happiness] * Gate [G500]	-0.04	-0.17 – 0.08	0.496	478.00
Emotion [Sadness] * Gate [G500]	-0.20	-0.33 – -0.08	**0.002**	478.00
Emotion [Fear] * Gate [G600]	-0.12	-0.25 – 0.00	0.057	478.00
Emotion [Happiness] * Gate [G600]	-0.02	-0.14 – 0.11	0.775	478.00
Emotion [Sadness] * Gate [G600]	-0.16	-0.29 – -0.03	**0.013**	478.00
Emotion [Fear] * Gate [GFULL]	-0.01	-0.14 – 0.11	0.831	478.00
Emotion [Happiness] * Gate [GFULL]	0.22	0.09 – 0.35	**0.001**	478.00
Emotion [Sadness] * Gate [GFULL]	-0.17	-0.30 – -0.05	**0.008**	478.00

Random Effects

σ^2	0.03
$\tau_{00\ Subject}$	0.00
ICC	0.14
$N_{Subject}$	25
Observations	500
Marginal R^2 / Conditional R^2	0.416 / 0.499

Post hoc test results of LMM S4A (Arab group)

contrast	Emotion	estimate	*SE*	*df*	*t*	*p*
G200 - G400	Anger	-0.16	0.05	475.00	-3.42	.006**
G200 - G500	Anger	-0.19	0.05	475.00	-4.25	< .001***
G200 - G600	Anger	-0.19	0.05	475.00	-4.22	< .001***
G200 - GFULL	Anger	-0.39	0.05	475.00	-8.62	< .001***
G400 - G500	Anger	-0.04	0.05	475.00	-0.83	.922
G400 - G600	Anger	-0.04	0.05	475.00	-0.80	.930
G400 - GFULL	Anger	-0.24	0.05	475.00	-5.20	< .001***

Post hoc test results of LMM S4A (Arab group)

contrast	Emotion	estimate	*SE*	*df*	*t*	*p*
G500 - G600	Anger	0.00	0.05	475.00	0.03	1.00
G500 - GFULL	Anger	-0.20	0.05	475.00	-4.37	< .001***
G600 - GFULL	Anger	-0.20	0.05	475.00	-4.40	< .001***
G200 - G400	Fear	-0.07	0.05	475.00	-1.60	.500
G200 - G500	Fear	-0.03	0.05	475.00	-0.72	.952
G200 - G600	Fear	-0.07	0.05	475.00	-1.52	.547
G200 - GFULL	Fear	-0.38	0.05	475.00	-8.32	< .001***
G400 - G500	Fear	0.04	0.05	475.00	0.88	.906
G400 - G600	Fear	0.00	0.05	475.00	0.07	1.00
G400 - GFULL	Fear	-0.31	0.05	475.00	-6.72	< .001***
G500 - G600	Fear	-0.04	0.05	475.00	-0.80	.930
G500 - GFULL	Fear	-0.35	0.05	475.00	-7.60	< .001***

Post hoc test results of LMM S4A (Arab group)

contrast	Emotion	estimate	*SE*	*df*	*t*	*p*
G600 - GFULL	Fear	-0.31	0.05	475.00	-6.79	< .001***
G200 - G400	Happiness	-0.15	0.05	475.00	-3.28	.010**
G200 - G500	Happiness	-0.15	0.05	475.00	-3.28	.010**
G200 - G600	Happiness	-0.17	0.05	475.00	-3.81	.001**
G200 - GFULL	Happiness	-0.61	0.05	475.00	-13.48	< .001***
G400 - G500	Happiness	-0.00	0.05	475.00	-0.00	1.00
G400 - G600	Happiness	-0.02	0.05	475.00	-0.53	.984
G400 - GFULL	Happiness	-0.46	0.05	475.00	-10.20	< .001***
G500 - G600	Happiness	-0.02	0.05	475.00	-0.53	.984
G500 - GFULL	Happiness	-0.46	0.05	475.00	-10.19	< .001***
G600 - GFULL	Happiness	-0.44	0.05	475.00	-9.66	< .001***
G200 - G400	Sadness	-0.02	0.05	475.00	-0.48	.989

Post hoc test results of LMM S4A (Arab group)

contrast	Emotion	estimate	*SE*	*df*	*t*	*p*
G200 - G500	Sadness	0.01	0.05	475.00	0.23	.999
G200 - G600	Sadness	-0.03	0.05	475.00	-0.69	.959
G200 - GFULL	Sadness	-0.22	0.05	475.00	-4.82	< .001***
G400 - G500	Sadness	0.03	0.05	475.00	0.72	.952
G400 - G600	Sadness	-0.01	0.05	475.00	-0.20	1.00
G400 - GFULL	Sadness	-0.20	0.05	475.00	-4.34	< .001***
G500 - G600	Sadness	-0.04	0.05	475.00	-0.92	.888
G500 - GFULL	Sadness	-0.23	0.05	475.00	-5.06	< .001***
G600 - GFULL	Sadness	-0.19	0.05	475.00	-4.14	< .001***

S4B – Analysis of recognition latency (Emotion Identification Poiunts) for native speech prosody

LMM S4B (*EIPtime (Native prosody) ~ Group + Emotion + Group*Emotion + GFullDuration + (1 | Subject))* and post hoc tests showing latency of recognition for native prosody by Group (Chinese, Arab) and emotion type (anger, fear, happiness, sadness).

**EIP as a function of native prosody and Group**				
Predictors	*Estimates*	*CI*	*p*	*df*
(Intercept)	223.39	90.78 – 355.99	**0.001**	1753.00
Group [Chinese]	-343.96	-464.59 – -223.33	**<0.001**	1753.00
Emotion [Fear]	148.49	49.40 – 247.59	**0.003**	1753.00
Emotion [Happiness]	196.93	102.25 – 291.60	**<0.001**	1753.00
Emotion [Sadness]	91.69	-13.10 – 196.48	0.086	1753.00
FullDuration ms	0.39	0.31 – 0.47	**<0.001**	1753.00
Group [Chinese] × Emotion [Fear]	153.44	18.77 – 288.12	**0.026**	1753.00
Group [Chinese] × Emotion [Happiness]	462.45	332.47 – 592.44	**<0.001**	1753.00
Group [Chinese] × Emotion [Sadness]	-242.45	-380.12 – -104.77	**0.001**	1753.00
**Random Effects**				
σ^2	242744.02			
$\tau_{00\ Subject}$	17746.37			
ICC	0.07			

$N_{Subject}$	50
Observations	1764
Marginal R^2 / Conditional R^2	0.223 / 0.276

Post hoc test results of LMM S4B (divided by Emotion)

contrast	Emotion	estimate	*SE*	*df*	*t*	*p*
Arabic - Chinese	Anger	343.96	61.51	153.96	5.59	< .001***
Arabic - Chinese	Fear	190.52	62.33	162.26	3.06	.003**
Arabic - Chinese	Happiness	-118.49	59.60	135.95	-1.99	.049*
Arabic - Chinese	Sadness	586.41	64.43	182.53	9.10	< .001***

Post hoc test results of LMM S4B (divided by Group)

contrast	Group	estimate	*SE*	*df*	*t*	*p*
Anger - Fear	Arabic	-148.49	50.52	1,735.33	-2.94	.018*
Anger - Happiness	Arabic	-196.93	48.27	1,718.51	-4.08	< .001***

Post hoc test results of LMM S4B (divided by Group)

contrast	Group	estimate	*SE*	*df*	*t*	*p*
Anger - Sadness	Arabic	-91.69	53.43	1,740.29	-1.72	.316
Fear - Happiness	Arabic	-48.43	49.21	1,734.12	-0.98	.759
Fear - Sadness	Arabic	56.81	54.58	1,754.27	1.04	.725
Happiness - Sadness	Arabic	105.24	49.73	1,744.46	2.12	.148
Anger - Fear	Chinese	-301.94	46.25	1,716.77	-6.53	< .001***
Anger - Happiness	Chinese	-659.38	48.21	1,723.42	-13.68	< .001***
Anger - Sadness	Chinese	150.76	51.21	1,713.98	2.94	.017*
Fear - Happiness	Chinese	-357.44	46.87	1,722.77	-7.63	< .001***
Fear - Sadness	Chinese	452.70	48.96	1,717.05	9.25	< .001***
Happiness - Sadness	Chinese	810.14	45.92	1,721.50	17.64	< .001***
