# Supplementary material for: Speak or shout? Nonverbal vocalizations promote rapid detection of emotions in vocal communication
Source: PLoS One. 2026 Jan 8;21(1):e0327529. doi: 10.1371/journal.pone.0327529 (PMC12782396; doi:10.1371/journal.pone.0327529)
Supplement: S5 Table — (PDF) [file pone.0327529.s005.pdf]

S5 Table. Statistical results of models comparing vocalizations and native speech prosody by Group, averaged across emotions, for A) recognition accuracy (Hu scores) and B) recognition latency (Emotion Identification Points).

S5A – Analysis of recognition accuracy (Hu scores) by Group and Event type

LMM *(HuScore (GFull) ~ Group + EventType + Group*EventType + (1|Subject) + (1|Emotion))* and post hoc tests showing recognition accuracy by Group and Event type.

Predictors	*Estimates*	*CI*	*p*	*df*
(Intercept)	0.57	0.50 – 0.63	**<0.001**	393.00
Group [Chinese]	0.06	-0.03 – 0.14	0.188	393.00
EventType [Vocalization]	0.15	0.10 – 0.20	**<0.001**	393.00
Group [Chinese] × EventType [Vocalization]	-0.11	-0.18 – -0.04	**0.002**	393.00
**Random Effects**				
σ^2	0.03			
$\tau_{00\ Subject}$	0.02			
$\tau_{00\ Emotion}$	0.00			
ICC	0.34			
N Subject	50			
N Emotion	4			
Observations	400			
Marginal R^2 / Conditional R^2	0.056 / 0.375			

Post hoc test results of LMM S5A

contrast	Group	estimate	*SE*	*df*	*t*	*p*
Vocalization – Speech	Arab	0.15	0.03	346.60	5.83	< .001***
Vocalization - Speech	Chinese	0.03	0.03	346.60	1.38	.169

Post hoc test results of LMM 5A

contrast	EventType	estimate	*SE*	*df*	*t*	*p*
Chinese – Arab	Speech	0.06	0.04	71.41	1.32	.191
Chinese – Arab	Vocalization	-0.05	0.04	71.41	-1.24	.218

S5B – Analysis of recognition latency (Emotion Identification Points) by Group and Event type

LMM *(EIPtime ~ Group + EventType + Group*EventType + GFullDuration + (1|Subject)) + (1 | Emotion)* and post hoc tests showing recognition latency by Group and Event type.

EIP as a function of Group x Event type

Predictors	*Estimates*	*CI*	*p*	*df*
(Intercept)	599.07	470.05 – 728.08	**<0.001**	3510.00
Group [Chinese]	-207.44	-285.08 – -129.81	**<0.001**	3510.00
EventType [Vocalization]	-474.88	-518.33 – -431.43	**<0.001**	3510.00
FullDuration ms	0.19	0.14 – 0.24	**<0.001**	3510.00
Group [Chinese] × EventType [Vocalization]	255.60	193.76 – 317.45	**<0.001**	3510.00
**Random Effects**				
σ^2	207737.59			
$\tau_{00\ Subject}$	12994.43			
$\tau_{00\ Emotion}$	9457.14			
ICC	0.10			
$N_{Subject}$	50			
$N_{Emotion}$	4			
Observations	3518			
Marginal R^2 / Conditional R^2	0.153 / 0.236			

Post hoc test results of LMM S5B (divided by Event type)

contrast	ItemType	estimate	*SE*	*df*	*t*	*p*
Chinese - Arabic	Speech	-207.44	39.60	60.19	-5.24	< .001***
Chinese - Arabic	Vocalization	48.16	38.99	56.67	1.24	.222

Post hoc test results of LMM S5B (divided by Group)

contrast	Group	estimate	*SE*	*df*	*t*	*p*
Vocalization - Speech	Arabic	-474.88	22.16	3,463.40	-21.43	< .001***
Vocalization - Speech	Chinese	-219.28	22.32	3,466.62	-9.82	< .001***
