# Supplementary material for: Speak or shout? Nonverbal vocalizations promote rapid detection of emotions in vocal communication
Source: PLoS One. 2026 Jan 8;21(1):e0327529. doi: 10.1371/journal.pone.0327529 (PMC12782396; doi:10.1371/journal.pone.0327529)
Supplement: S6 Table — (PDF) [file pone.0327529.s006.pdf]

S6 Table. Statistical results of models comparing emotional prosody recognition by language for A-B) accuracy (Hu scores) and C) latency (Emotion Identification Points).

S6A – Analysis of recognition accuracy (Hu scores) by language familiarity and gate duration, separately by Group

LMM (*HuScore ~ (Familiarity + Gate + Familiarity*Gate + (1 | Subject) + (1 | Emotion*)) and post hoc tests showing recognition accuracy by Group and event type.

Chinese Group LMM

Predictors	*Estimates*	*CI*	*p*	*df*
(Intercept)	0.11	0.04 – 0.18	**0.003**	1482.00
Familiarity [Native]	0.21	0.16 – 0.25	**<0.001**	1482.00
Familiarity [L2-English]	-0.02	-0.07 – 0.02	0.320	1482.00
Gate [G400]	0.05	0.01 – 0.10	**0.016**	1482.00
Gate [G500]	0.08	0.04 – 0.12	**<0.001**	1482.00
Gate [G600]	0.09	0.04 – 0.13	**<0.001**	1482.00
Gate [GFULL]	0.28	0.24 – 0.33	**<0.001**	1482.00
Familiarity [Native] * Gate [G400]	0.01	-0.05 – 0.07	0.711	1482.00
Familiarity [L2-English] * Gate [G400]	-0.00	-0.06 – 0.06	0.987	1482.00
Familiarity [Native] * Gate [G500]	-0.00	-0.06 – 0.06	0.985	1482.00

Familiarity [L2-English] * Gate [G500]	0.02	-0.05 – 0.08	0.593	1482.00
Familiarity [Native] * Gate [G600]	0.02	-0.04 – 0.09	0.461	1482.00
Familiarity [L2-English] * Gate [G600]	0.04	-0.02 – 0.11	0.158	1482.00
Familiarity [Native] * Gate [GFULL]	0.02	-0.04 – 0.08	0.517	1482.00
Familiarity [L2-English] * Gate [GFULL]	0.01	-0.06 – 0.07	0.846	1482.00
**Random Effects**				
σ^2	0.03			
$\tau_{00\ Subject}$	0.01			
$\tau_{00\ Emotion}$	0.00			
ICC	0.27			
$N_{Subject}$	25			
$N_{Emotion}$	4			
Observations	1500			
Marginal R^2 / Conditional R^2	0.379 / 0.548			

Post hoc test results of LMM S6A (Chinese group)

contrast	estimate	*SE*	*df*	*t*	*p*
Native - Foreign	0.22	0.01	1,471.97	21.91	< .001***
L2-English - Foreign	-0.01	0.01	1,471.97	-0.88	.654
L2-English - Native	-0.23	0.01	1,471.97	-22.78	< .001***
G400 - G200	0.06	0.01	1,471.97	4.48	< .001***
G500 - G200	0.09	0.01	1,471.97	6.67	< .001***
G500 - G400	0.03	0.01	1,471.97	2.19	.185
G600 - G200	0.11	0.01	1,471.97	8.42	< .001***
G600 - G400	0.05	0.01	1,471.97	3.94	.001***
G600 - G500	0.02	0.01	1,471.97	1.75	.403
GFULL - G200	0.29	0.01	1,471.97	22.74	< .001***
GFULL - G400	0.24	0.01	1,471.97	18.26	< .001***
GFULL - G500	0.21	0.01	1,471.97	16.07	< .001***
GFULL - G600	0.18	0.01	1,471.97	14.32	< .001***

Arab Group LMM

Predictors	*Estimates*	*CI*	*p*	*df*
(Intercept)	0.28	0.19 – 0.38	**<0.001**	1482.00
Familiarity [Native]	-0.12	-0.17 – -0.07	**<0.001**	1482.00
Familiarity [L2-English]	-0.16	-0.21 – -0.11	**<0.001**	1482.00
Gate [G400]	0.08	0.03 – 0.13	**0.001**	1482.00
Gate [G500]	0.11	0.06 – 0.15	**<0.001**	1482.00
Gate [G600]	0.12	0.08 – 0.17	**<0.001**	1482.00
Gate [GFULL]	0.26	0.22 – 0.31	**<0.001**	1482.00
Familiarity [Native] * Gate [G400]	0.02	-0.05 – 0.09	0.554	1482.00
Familiarity [L2-English] * Gate [G400]	0.02	-0.05 – 0.09	0.610	1482.00
Familiarity [Native] * Gate [G500]	-0.01	-0.08 – 0.05	0.686	1482.00
Familiarity [L2-English] * Gate [G500]	0.04	-0.03 – 0.11	0.219	1482.00
Familiarity [Native] * Gate [G600]	-0.01	-0.08 – 0.06	0.807	1482.00
Familiarity [L2-English] * Gate [G600]	0.07	0.01 – 0.14	**0.032**	1482.00
Familiarity [Native] * Gate [GFULL]	0.14	0.07 – 0.20	**<0.001**	1482.00

Familiarity [L2-English] * Gate [GFULL]	0.17	0.10 – 0.24	**<0.001**	1482.00

**Random Effects**	
σ^2	0.03
$\tau_{00\ Subject}$	0.01
$\tau_{00\ Emotion}$	0.01
ICC	0.28
$N_{Subject}$	25
$N_{Emotion}$	4
Observations	1500
Marginal R^2 / Conditional R^2	0.300 / 0.497

Post hoc test results of LMM S6A (Arab group)

contrast	Familiarity	estimate	*SE*	*df*	*t*	*p*
G400 - G200	Foreign	0.08	0.02	1,471.98	3.26	.010**
G500 - G200	Foreign	0.11	0.02	1,471.98	4.32	< .001***
G500 - G400	Foreign	0.03	0.02	1,471.98	1.05	.830
G600 - G200	Foreign	0.12	0.02	1,471.98	5.13	< .001***

Post hoc test results of LMM S6A (Arab group)

contrast	Familiarity	estimate	*SE*	*df*	*t*	*p*
G600 - G400	Foreign	0.05	0.02	1,471.98	1.87	.335
G600 - G500	Foreign	0.02	0.02	1,471.98	0.81	.926
GFULL - G200	Foreign	0.26	0.02	1,471.98	10.87	< .001***
GFULL - G400	Foreign	0.19	0.02	1,471.98	7.61	< .001***
GFULL - G500	Foreign	0.16	0.02	1,471.98	6.55	< .001***
GFULL - G600	Foreign	0.14	0.02	1,471.98	5.74	< .001***
G400 - G200	Native	0.10	0.02	1,471.98	4.10	< .001***
G500 - G200	Native	0.09	0.02	1,471.98	3.74	.002**
G500 - G400	Native	-0.01	0.02	1,471.98	-0.36	.997
G600 - G200	Native	0.12	0.02	1,471.98	4.78	< .001***
G600 - G400	Native	0.02	0.02	1,471.98	0.68	.960
G600 - G500	Native	0.03	0.02	1,471.98	1.04	.836

Post hoc test results of LMM S6A (Arab group)

contrast	Familiarity	estimate	*SE*	*df*	*t*	*p*
GFULL - G200	Native	0.40	0.02	1,471.98	16.46	< .001***
GFULL - G400	Native	0.30	0.02	1,471.98	12.36	< .001***
GFULL - G500	Native	0.31	0.02	1,471.98	12.72	< .001***
GFULL - G600	Native	0.28	0.02	1,471.98	11.67	< .001***
G400 - G200	L2-English	0.10	0.02	1,471.98	3.98	.001***
G500 - G200	L2-English	0.15	0.02	1,471.98	6.05	< .001***
G500 - G400	L2-English	0.05	0.02	1,471.98	2.07	.233
G600 - G200	L2-English	0.20	0.02	1,471.98	8.17	< .001***
G600 - G400	L2-English	0.10	0.02	1,471.98	4.18	< .001***
G600 - G500	L2-English	0.05	0.02	1,471.98	2.11	.216
GFULL - G200	L2-English	0.43	0.02	1,471.98	17.77	< .001***
GFULL - G400	L2-English	0.34	0.02	1,471.98	13.79	< .001***

Post hoc test results of LMM S6A (Arab group)						
contrast	Familiarity	estimate	*SE*	*df*	*t*	*p*
GFULL - G500	L2-English	0.29	0.02	1,471.98	11.72	< .001***
GFULL - G600	L2-English	0.23	0.02	1,471.98	9.61	< .001***

S6B – Analysis of recognition accuracy (Hu scores) by Group and recorded language (Mandarin, Arabic, English).

LMM (*HuScore (GFull) ~ (Language + Group + Language* Group + (1 | Subject) + (1 | Emotion*)) and post hoc tests showing recognition accuracy by recorded language (Mandarin, Arabic, English) and Group (Chinese, Arab).

Predictors	*Estimates*	*CI*	*p*	*df*
(Intercept)	0.57	0.49 – 0.64	**<0.001**	591.00
Language [English]	-0.01	-0.06 – 0.04	0.740	591.00
Language [Mandarin]	-0.02	-0.07 – 0.04	0.543	591.00
Group [Chinese]	-0.17	-0.26 – -0.08	**<0.001**	591.00
Language [English] * Group [Chinese]	-0.01	-0.08 – 0.07	0.849	591.00
Language [Mandarin] * Group [Chinese]	0.24	0.17 – 0.32	**<0.001**	591.00

**Random Effects**	
σ^2	0.04

$\tau_{00\ Subject}$	0.02
$\tau_{00\ Emotion}$	0.00
ICC	0.32
$N_{Subject}$	50
$N_{Emotion}$	4
Observations	600
Marginal R^2 / Conditional R^2	0.137 / 0.414

Post hoc test results of LMM S6B

contrast	Group	estimate	*SE*	*df*	*t*	*p*
English - Arabic	Arab	-0.01	0.03	546.82	-0.33	.941
Mandarin - Arabic	Arab	-0.02	0.03	546.82	-0.61	.816
Mandarin - English	Arab	-0.01	0.03	546.82	-0.28	.959
English - Arabic	Chinese	-0.02	0.03	546.82	-0.60	.820
Mandarin - Arabic	Chinese	0.23	0.03	546.82	8.54	< .001***
Mandarin – English	Chinese	0.24	0.03	546.82	9.14	< .001***
contrast	Language	estimate	*SE*	*df*	*t*	*p*
Chinese - Arab	Arabic	-0.17	0.04	85.93	-3.84	< .001***

Post hoc test results of LMM S6B						
contrast	Group	estimate	*SE*	*df*	*t*	*p*
Chinese - Arab	English	-0.18	0.04	85.93	-4.01	< .001***
Chinese - Arab	Mandarin	0.07	0.04	85.93	1.67	.098

S6C – Analysis of recognition latency (Emotion Identification Points) by Group and language familiarity

LMM (*EIPtime ~ (Group + Familiarity + Group*Familiarity + GFullDuration + (1 | Subject) + (1 | Emotion*)) and post hoc tests showing latency of emotional prosody recognition by Group (Chinese, Arab) and language familiarity (native, L2-English, foreign)

EIP as a function of Group and Language familiarity (S6C)				
Predictors	*Estimates*	*CI*	*p*	*df*
(Intercept)	283.01	71.88 – 494.14	**0.009**	4769.00
Group [Chinese]	361.09	280.03 – 442.15	**<0.001**	4769.00
Familiarity [L1]	272.45	219.03 – 325.88	**<0.001**	4769.00
Familiarity [L2]	226.99	174.31 – 279.67	**<0.001**	4769.00
FullDuration ms	0.21	0.16 – 0.26	**<0.001**	4769.00
Group [Chinese] × Familiarity [L1]	-569.27	-648.74 – -489.79	**<0.001**	4769.00
Group [Chinese] × Familiarity [L2]	-206.28	-286.11 – -126.44	**<0.001**	4769.00

**Random Effects**	
σ^2	296343.46
$\tau_{00\ Subject}$	10404.44
$\tau_{00\ Emotion}$	37650.26
ICC	0.14
$N_{Subject}$	50
$N_{Emotion}$	4
Observations	4779
Marginal R^2 / Conditional R^2	0.074 / 0.203

Post hoc test results of LMM S6C

contrast	Group	estimate	*SE*	*df*	*t*	*p*
Native - Foreign	Arab	272.45	27.25	4,726.91	10.00	< .001***
L2-English - Foreign	Arab	226.99	26.87	4,727.93	8.45	< .001***
L2-English - Native	Arab	-45.46	28.60	4,726.47	-1.59	.250
Native - Foreign	Chinese	-296.81	29.00	4,747.06	-10.24	< .001***
L2-English - Foreign	Chinese	20.71	32.35	4,733.38	0.64	.798
L2-English - Native	Chinese	317.53	27.88	4,733.08	11.39	< .001***

Post hoc test results of LMM S6C

contrast	Group	estimate	*SE*	*df*	*t*	*p*
contrast	Familiarity	estimate	*SE*	*df*	*t*	*p*
Chinese - Arab	Foreign	361.09	41.35	100.13	8.73	< .001***
Chinese - Arab	Native	-208.18	39.44	83.55	-5.28	< .001***
Chinese - Arab	L2-English	154.81	40.49	92.41	3.82	< .001***
